# A natural uORF variant confers phosphorus acquisition diversity in soybean

**DOI:** 10.1038/s41467-022-31555-2

**Published:** 2022-07-01

**Authors:** Zilong Guo, Hongrui Cao, Jing Zhao, Shuang Bai, Wenting Peng, Jian Li, Lili Sun, Liyu Chen, Zhihao Lin, Chen Shi, Qing Yang, Yongqing Yang, Xiurong Wang, Jiang Tian, Zhichang Chen, Hong Liao

**Affiliations:** 1 Root Biology Center, Fujian Agriculture and Forestry University, Fuzhou, China; 2 Root Biology Center, South China Agricultural University, Guangzhou, China

**Keywords:** Natural variation in plants, Agricultural genetics, Abiotic

## Abstract

Phosphorus (P) is an essential element for all organisms. Because P fertilizers are a non-renewable resource and high fixation in soils, sustainable agriculture requires researchers to improve crop P acquisition efficiency. Here, we report a strong association signal at a locus of *CPU1* (*component of phosphorus uptake 1*), from a genome-wide association study of P acquisition efficiency in a soybean core collection grown in the field. A SEC12-like gene, *GmPHF1*, is identified as the causal gene for *CPU1*. GmPHF1 facilitates the ER (endoplasmic reticulum) exit of the phosphate transporter, GmPT4, to the plasma membrane of root epidermal cells. A common SNP in an upstream open reading frame (uORF) of *GmPHF1*, which alters the abundance of GmPHF1 in a tissue-specific manner, contributes to P acquisition diversity in soybean. A natural genetic variation conditions diversity in soybean P acquisition, which can be used to develop P-efficient soybean genotypes.

## Abstract (web summary)

Improving phosphorus (P) acquisition efficiency is important for sustainable agriculture. Here, the authors report a natural variation in an upstream open reading frame of a *SEC12-like* gene *GmPHF1* is crucial for protein abundance and spatial distribution of GmPHF1, which contribute to P acquisition diversity in soybean.

## Introduction

As a non-renewable resource, phosphorus (P) is one of the most important essential macronutrients for plant growth and development^1^. However, high P-retention in soils results in P deficiency for most crops^2^, and high rates of P fertilizers have to be applied into agroecosystems to maintain crop yield. The high input of chemical P fertilizers not only increases the financial burden to farmers but also causes environmental risks^3^. Therefore, genetic manipulation to improve crop P-acquisition efficiency is a highly valued goal for more sustainable agriculture.

Understanding the molecular mechanisms and genetic bases underlying crop P acquisition can help facilitate the breeding of P-efficient crops. Functional genes involved in root morphology and architecture^4^, phosphate (Pi) transport^5^, as well as metabolism and transport of root exudates^6^, have been identified and can contribute to root P acquisition. On the other hand, many efforts on exploring P acquisition by forward genetic approaches have been made^7,8^; however, little success was achieved. Rice *PSTOL1* (*phosphorus-starvation tolerance 1*) was a P-efficiency gene identified via QTL mapping of tiller number, P uptake, and biomass production under low P conditions^9–11^. Subsequently, Hufnagel et al. conducted candidate gene-association analysis for sorghum P efficiency via both field and lab phenotyping and showed that several sorghum *PSTOL1* homologs condition P efficiency by enhancing shallow lateral root growth^12^. Nevertheless, other loci responsible for P acquisition beyond root traits remain largely unknown.

Soybean (*Glycine max*) is a globally important legume crop providing proteins and oils for human and animals^13^. The high seed protein and oil content in soybean^13^ are indicative of its high demand for nutrients from the environment. Since soybean can fix atmospheric nitrogen (N) through symbiosis with microorganisms to meet its N requirement^14^, soil P becomes the maximum constraint for soybean growth.

In this work, to investigate traits that could enhance soil P acquisition in soybean, a field experiment is performed using our previously-constructed soybean core collection containing candidate P-efficiency genotypes^15^. Combining millions of SNPs based on whole-genome re-sequencing, GWAS is conducted to explore the genetic bases for P acquisition in soybean, and a strong association signal on chromosome 20 is detected. A naturally occurring variant in an upstream open reading frame (uORF) is found to be crucial for protein abundance and spatial distribution of the downstream causal gene, which consequently contributes to P acquisition diversity in soybean.

## Results

### Re-sequencing and characterization of an applied soybean core collection

**Fig. 1:**
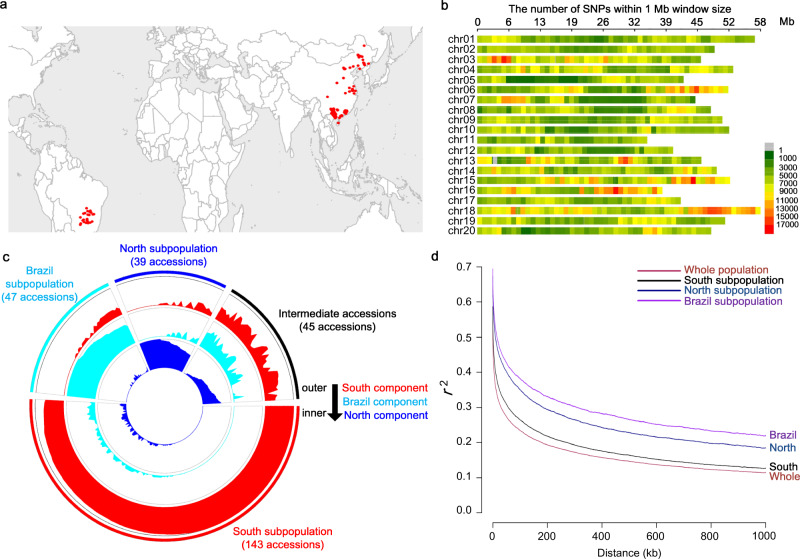
Characterizing a P-efficiency applied core collection of soybean based on whole-genome re-sequencing. **a** Geographical distribution of re-sequenced soybean accessions. Each red dot indicates an accession. **b** SNP density along 20 chromosomes. The color indicates the number of SNPs in a 1-Mb region. **c** Percentage of inferred subpopulation components harbored by each accession using ADMIXTURE software under an assumption of ancient cluster *K* = 3. Red, cyan, and blue regions indicate South, Brazil, and North subpopulation components, respectively. The line height indicates the percentage of inferred subpopulation components. **d** LD levels indicated by *r*^2^ along physical distance in the whole population, South, Brazil, and North subpopulations, respectively. Source data underlying panel c are provided as a Source Data file.

We constructed an applied core collection for P efficiency from soybean germplasm in previous work^15^. In the population, a total of 274 accessions were collected using a GIS (geographical information system)-assisted approach based on soil types and soil P status of soybean production areas and agronomic traits of soybean^15,16^, which were mainly distributed in three geographical regions: South China, North China, and Brazil (Fig. 1a). In the study, we re-sequenced the whole genomes of 274 accessions and obtained approximately 2 terabases of DNA sequences from 13.5 billion reads after quality control, with an average depth ~7.4× for each accession (Supplementary Data 1). After alignment to the soybean reference genome (*Glycine ma*x Wm82.v2.0.40 from EnsemblPlants), followed by variant calling, low-quality variant filtering, and genotype imputation, a total of 6,300,394 high-quality bi-allelic SNPs with 1 SNP per 151 bp on average (Fig. 1b and Supplementary Table 1), and 2,117,569 insertions/deletions (INDELs) were identified (Supplementary Table 2).

Based on the genotypes of genome-wide SNP sites, we inferred the population structure and phylogenetic relationships for the core collection and found three distinct groups, which corresponded to three geographical distributions (Fig. 1c and Supplementary Fig. 1). Based on the geographical origins of the accessions within each group, the three distinct groups were termed “South” subpopulation (from South China), “Brazil” subpopulation (from Brazil), and “North” subpopulation (from North China), respectively; the remaining 45 accessions showed mixed subpopulation components, which were termed “intermediate” accessions (Fig. 1c and Supplementary Data 1). While there was a modest population differentiation for South-Brazil (*F*_ST_ = 0.222), relatively high levels of population differentiation were observed for South-North (*F*_ST_ = 0.365) and Brazil-North (*F*_ST_ = 0.374). To identify divergent genomic regions underlying the subpopulation differentiation, we performed genome-wide population differentiation analyses (*F*_ST_) between subpopulations. In total, 51, 47, and 9 divergent regions (*F*_ST_ ≥ 0.65 (top 5% *F*_ST_)) were identified for *Brazil-North*, *South-North*, and *South-Brazil*, respectively (Supplementary Fig. 2 and Supplementary Data 2).

To evaluate the mapping resolution of the genome-wide association study (GWAS), we estimated the genome-wide linkage disequilibrium (LD) levels. LD decayed along with the extension of physical distance and the decay rates varied among different (sub)populations. LD decayed much more rapidly in the whole population and the South subpopulation than in the North and Brazil subpopulations, which may be partially attributed to larger sample size (Fig. 1d). In addition, the LD-decay rates of different chromosomes varied in the same population (Supplementary Fig. 3). These results suggest that GWAS could provide relatively high mapping resolution to identify candidate genes in the whole population and the South subpopulation.

To evaluate the performance of GWAS using this soybean population, we performed a GWAS of flower color using linear mixed models (LMM) in the whole population (Supplementary Fig. 4). A strong and clear association signal on chromosome 13 was detected and the lead SNP sf1317311391 (*P*_LMM_ = 6.51 × 10^−81^) was approximately 1 kb away from *W1* (*Glyma13G072100*), an a priori gene controlling flower color in soybean^17^. The quantile–quantile plot showed a good performance in controlling *P* value inflations.

### A major locus *CPU1* associated with P-acquisition efficiency

**Fig. 2:**
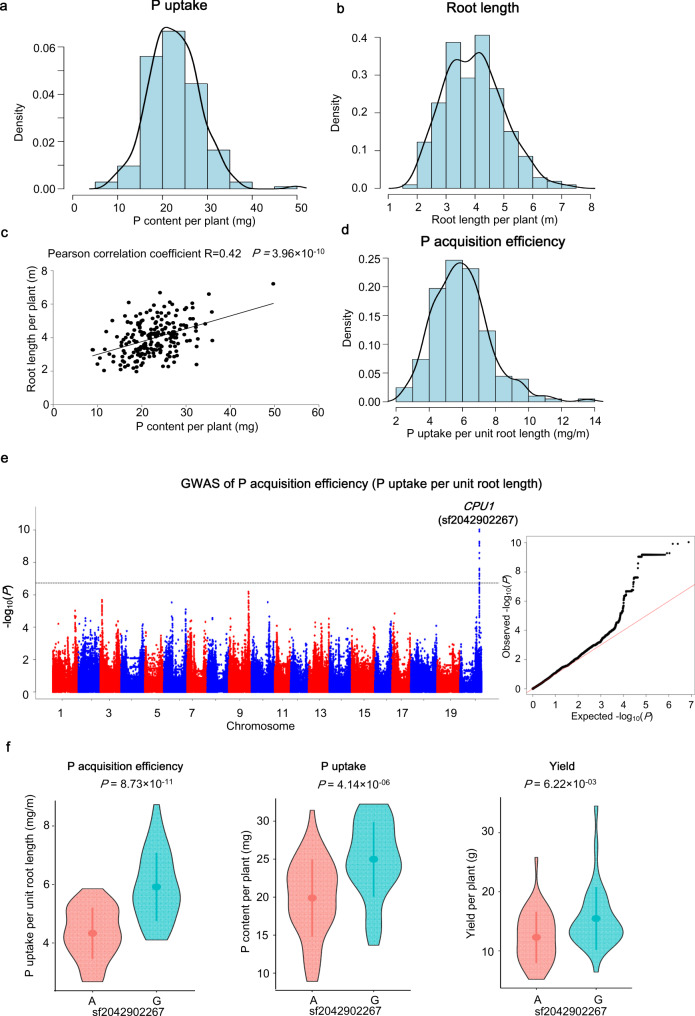
GWAS of P-acquisition efficiency in the natural population of soybean in the field. **a** Histogram and density plot showing phenotypic distributions of plant P uptake. **b** Histogram and density plot showing phenotypic distributions of total root length. **c** Scatter plot showing phenotypic correlation of P uptake and total root length. Pearson correlation coefficient is shown and *P* value was calculated based on two-sided *t*-test. **d** Histogram and density plot showing phenotypic distributions of P-acquisition efficiency (P uptake per unit root length). Following a randomized complete block design in the field, averaged values of plant P content and root length of four biological replications for each accession at the seedling stage (one month after sowing) were calculated. P uptake per unit root length was calculated by dividing P content by total root length. **e** Manhattan plot (left) and quantile–quantile plot (right) of P-acquisition efficiency (P uptake per unit root length). *n* = 4 biologically independent experiments following a randomized complete block design in the field. For Manhattan plot, −log_10_ *P* values are plotted against the position of SNPs on 20 chromosomes. Gray horizontal dashed line indicates genome-wide *P* value threshold determined by Bonferroni correction. The lead SNPs are shown above the corresponding association signals. For quantile–quantile plot, −log_10_-transformed observed *P* values are plotted against -log_10_-transformed expected *P* values. **f** Violin plots showing P-acquisition efficiency, P uptake, and yield of the two groups based on genotypes of the lead SNP sf2042902267. Points and error bars represent the averaged values and the standard deviation (SD) of each group, respectively. The *P* values were calculated using two-sided *t*-test. *n* = 49, 41; 52, 42; 40, 33 biologically independent samples, respectively. Source data underlying panels a–d, f are provided as a Source Data file.

Considering the close relationship between plant P uptake and root length^7,11^, we first determined plant P uptake (P content) and measured total root length, and then calculated P uptake per unit root length (P content/total root length) as a indicator of P-acquisition efficiency in 207 soybean accessions at the seedling stage (one month after sowing). There were large variations in both P uptake (ranged from 8.90 to 49.69 mg, Fig. 2a) and total root length (ranged from 1.97 to 7.20 m, Fig. 2b). Although the total root length was significantly correlated to the P uptake (Pearson correlation coefficient *R* = 0.42, *P* < 0.001) (Fig. 2c), the P uptake per unit root length for P-acquisition efficiency still showed large variation (ranged from 2.67 to 13.55 mg/m) (Fig. 2d), suggesting the existence of genetic loci contributing to P acquisition independently of root length.

In the study, we only performed GWAS in the whole population and South subpopulation due to the small sample size and small LD-decay rate of the North and Brazil subpopulations (Fig. 1d). The SNPs with a minor allele frequency (MAF) less than 0.05 were removed from the association analysis. A total of 4,618,874 and 3,757,697 SNPs remained for GWAS in the whole population and the South subpopulation, respectively (Supplementary Table 3). Using Bonferroni correction for multiple tests based on the number of effective independent SNPs to control the genetic type 1 error rate (Supplementary Table 3), we determined the genome-wide thresholds at 1.21 × 10^−7^ and 1.84 × 10^−7^ for the whole population and the South subpopulation, respectively. Adjacent SNPs having LD relationship with each other (*r*^2^ ≧ 0.30) were defined as a single locus to reduce the redundancy of association signals^18^. A SNP with the lowest *P* value for an association signal was defined as lead SNP.

GWAS of P uptake in the whole population and South subpopulation identified only one locus on chromosome 9 (*P*_LMM_ = 1.18 × 10^−7^ in the whole population; *P*_LMM_ = 6.56 × 10^−9^ in the South subpopulation) (Supplementary Fig. 5a and Supplementary Data 3). Even though the association signal failed to pass the genome-wide threshold, the locus was associated with plant biomass with the strongest signal (*P*_LMM_ = 1.73 × 10^−6^ in the whole population; *P*_LMM_ = 6.70 × 10^−7^ in the South subpopulation) (Supplementary Fig. 5b and Supplementary Data 3), suggesting that the locus at chromosome 9 may directly control plant growth but not P acquisition, and its statistical association with P acquisition may be partially attributed to the high correlation between plant biomass and plant P content in the mapping population (Pearson correlation coefficient *R* = 0.92). No significant loci were detected in the GWAS of total root length (Supplementary Fig. 5c). Strikingly, one strong association signal (named *CPU1* (*component of phosphorus uptake 1*)) on chromosome 20 was observed in the GWAS of P-acquisition efficiency using P uptake per unit root length as the indicator in the South subpopulation (*P*_LMM_ = 9.22 × 10^−11^) (Fig. 2e and Supplementary Data 3), and this locus was also detected for GWAS of P uptake when adjusting for root length (*P*_LMM_ = 1.17 × 10^−8^) (Supplementary Fig. 6b). For the lead SNP sf2042902267 of *CPU1*, the allele G of the lead SNP not only remarkably enhanced the value of P uptake per unit root length by 36.61% (*P* = 8.73 × 10^−11^, two-sided *t*-test), but also partly but significantly contributed to plant P uptake (*P* = 4.14 × 10^−06^, two-sided *t*-test) and yield (*P* = 6.22 × 10^−03^, two-sided *t*-test) (Fig. 2f), indicating the important role of this locus in both P acquisition and yield.

### Identification of the causal gene for *CPU1*

**Fig. 3:**
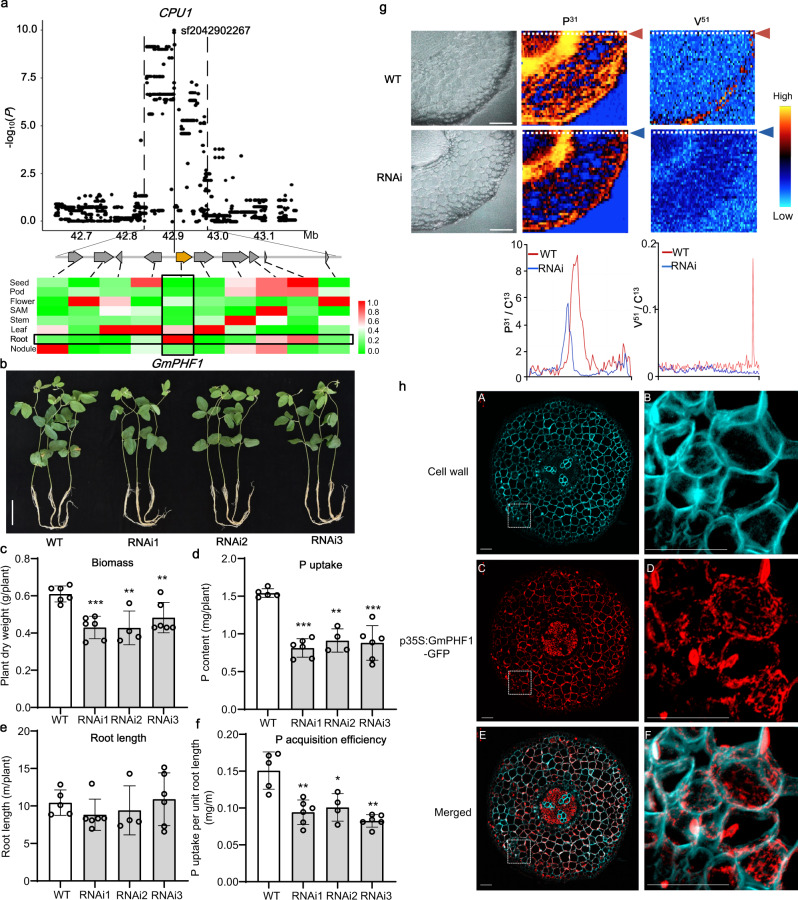
Functional characterization of GmPHF1 in P acquisition. **a** Local Manhattan plot and heatmap showing expression levels of candidate genes in multiple tissues. Before drawing the heatmap, the range of expression levels in multiple tissues for each candidate gene was transformed to 0–1 by linear normalization. **b** Growth of wild-type plant (WT) and three independent RNAi lines of *GmPHF1* at seedling stage (18-d-old plants). Bar = 10 cm. **c**–**f** Phenotypic parameters including plant biomass (**c**), P uptake (**d**), total root length (**e**), and P-acquisition efficiency (**f**) of the WT and three RNAi lines. Data are presented as mean values ± SD and asterisks indicate significance levels (***P < 0.001, **0.001 ≦*P* < 0.01, *0.01 ≦*P* < 0.05; two-sided *t*-test). For WT, RNAi1, RNAi2, and RNAi3, biologically independent sample size *n* = 6, 6, 4, 6 in **c**; *n* = 5, 6, 4, 6 in (**d**–**f**). *P* values are 1.40 × 10^−04^, 2.46 × 10^−03^, 7.29 × 10^−03^ in (**c**); 6.98 × 10^−07^, 2.10 × 10^−03^, 1.50 × 10^−04^ in (**d**); 2.02 × 10^−01^, 5.61 × 10^−01^, 7.94 × 10^−01^ in (**e**); 1.54 × 10^−03^, 1.34 × 10^−02^, 2.57 × 10^−03^ in (**f**). **g** LA-ICP-MS elemental mapping of root cross-section. P^31^ and V^51^ maps of root cross sections in WT (first row) and RNAi line (second row). The normalized element signals indicated by dotted lines in elemental maps were plotted (third row). Three independent biological replicates were investigated and a representative result was shown. Bars = 100 μm. **h** Immunostaining of p35S:GmPHF1-GFP transgenic hairy roots in soybean. Root cross sections were immunostained with a specific antibody against green fluorescent protein (GFP). Cyan shows signals from cell walls stained with Calcofluor-White (**A**, **B**), and red shows the anti-GFP signals (**C, D**). The merged image shows the combined two channels (**E**, **F**). White dotted areas in **A**, **C**, **E** are magnified in (**B**, **D**, **F**), respectively. Five independent transgenic lines were investigated and a representative result was shown. Scale bars are 50 μm. Source data underlying panels a and c–f are provided as a Source Data file.

To facilitate precise breeding, further work on exploring candidate gene for this locus was carried out. There were ten annotated genes in this P-acquisition-efficiency-associated locus *CPU1* (Supplementary Data 3), and the lead SNP sf2042902267 was closest to *Glyma.20G190300*, at a distance of 0.7 kb (Fig. 3a). *Glyma.20G190300* is primarily expressed in roots based on Phytozome database (https://phytozome.jgi.doe.gov/) and a soybean database of 1298 RNA-seq samples^19^ (https://venanciogroup.uenf.br/cgi-bin/gmax_atlas/index.cgi) (Fig. 3a and Supplementary Data 4). Furthermore, the gene’s expression was highly induced by P deficiency in basal roots (Supplementary Fig. 7). The gene contains 11 exons and 10 introns, encoding a peptide of 394 amino acids (https://phytozome.jgi.doe.gov/pz/portal.html). Based on phylogenetic analyses, the gene belongs to the plant SEC12-like protein family, and shows 66.67 and 57.59% protein sequence identity to AtPHF1 (*Arabidopsis thaliana* Phosphate Transporter Traffic Facilitator 1^20^,) and OsPHF1 (*Oryza sativa* Phosphate Transporter Traffic Facilitator 1^21^) (Supplementary Fig. 8), respectively. PHF1 was shown to interact with Pi transporters (PTs) and facilitate their trafficking to the plasma membrane (PM), and these interactions were repressed by PT phosphorylation^22^. We, therefore, designated this soybean gene *GmPHF1*, as the candidate of *CPU1*.

To investigate the function of GmPHF1 in P acquisition, three independent transgenic RNAi lines showing significantly lower *GmPHF1* expression levels were generated (Supplementary Fig. 9), and their phenotypes were compared with wild-type (WT) plants. At the seedling stage, three RNAi lines showed significant reductions in plant biomass, P uptake and P-acquisition efficiency (P uptake per unit root length), but showed little difference in total root length compared with WT (Fig. 3b–f), further confirming the GWAS result that *CPU1* contributes to P-acquisition independently of total root length. Analysis of the distribution of P in soybean roots was conducted using LA-ICP-MS. P^31^ maps of root cross-section showed that although P was preferentially accumulated at endodermis, WT exhibited much higher P signals than RNAi line (Fig. 3g). Given that vanadate (V) is a tracer for Pi uptake but not for translocation as V can be reduced to vanadyl once transported into cells, we further utilized V to monitor P uptake process in soybean roots. V^51^ maps of root samples after 6-h V feeding showed strong signals at root epidermal cells in WT, but not in RNAi line (Fig. 3g), suggesting the direct role of GmPHF1 in regulating P uptake. At the maturity stage, three RNAi lines presented much lower pod numbers than WT (Supplementary Fig. 10), suggesting the profound effects of GmPHF1 on later P acquisition and yield.

### GmPHF1-mediated P acquisition is GmPT4-dependent

AtPHF1 and OsPHF1 are localized in the endoplasmic reticulum (ER) and facilitate the ER exit of a phosphate transporter (PT)^20,21^. To determine the subcellular localization of the GmPHF1 protein, the *GmPHF1* ORF fused in frame with *GFP* was transiently expressed in tobacco leaves (*Nicotiana benthamiana* L.). The fluorescence signal of GFP (green color) was most pronounced in the endoplasmic reticulum (ER), which was co-located with the ER marker (CD3-960^23^,) (Supplementary Fig. 11). The subsequent immunostaining of transgenic hairy roots carrying *GmPHF1-GFP* further verified the ER localization pattern of GmPHF1 in soybean (Fig. 3h).

**Fig. 4:**
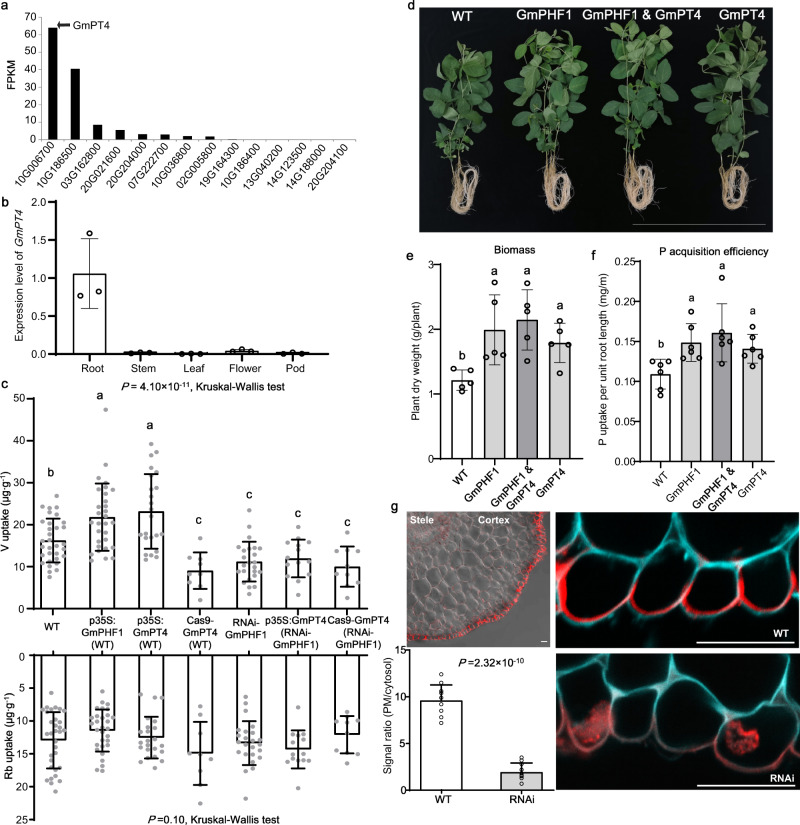
GmPHF1 interacts with GmPT4 for P uptake in soybean roots. **a** FPKM values of PT family genes in soybean roots (3-week-old) revealed by RNA-seq. **b** Expression levels of GmPT4 in different tissues of soybean revealed by real-time RT-PCR. Data are presented as mean values±SD of three biologically independent samples for each tissue. **c** V and Rb uptake assay in transgenic hairy roots. *p35S:GmPHF1*, *p35S:GmPT4*, and *Cas9-GmPT4* constructs were individually introduced into the hairy root of WT (YC04-5) or GmPHF1-RNAi line. The receptor parents of transgenic hairy root are indicated in parentheses. Root samples were treated with V (1 μM, Pi tracer) and Rb (1 μM, internal control) for 12 h. *P* values were calculated using Kruskal–Wallis test. Different letters indicate a significant difference in element uptake (*P* < 0.05). Data are presented as mean values ± SD. *n* = 32, 30, 23, 9, 24, 14, 10 biologically independent samples, respectively. **d** Growth of wild-type plant (WT), overexpression line of *GmPHF1 or GmPT4*, co-overexpression line of *GmPHF1* and *GmPT4* at seedling stage (20-d-old plants). Bar = 50 cm. **e**, **f** Phenotypic parameters including plant biomass (dry weight, (**e**)) and P-acquisition efficiency (P uptake per unit root length, (**f**)) of the WT and three overexpression lines in **d**. Data are presented as mean values ± SD. *n* = 5, 6 biologically independent samples in **e**, **f**, respectively. *P* values are 1.59 × 10^−02^ in **e** and 1.03 × 10^−02^ in **f** based on Kruskal–Wallis test and different letters indicate significant differences (*P* < 0.05). **g** Immunostaining of GmPT4 localization in WT and *GmPHF1*-RNAi line. Immunostaining was performed with *ProPT4: gPT4-GFP* transgenic hairy roots. Red fluorescence is the signal from the GFP antibody and Cyan fluorescence is the signal from the cell wall. Bars = 20 μm. Data are presented as mean values + SD of ten biological replications. *P* value was calculated based on two-sided *t*-test. Source data underlying panels a–c, e–g are provided as a Source Data file.

We next examined whether GmPHF1 affects Pi transporters in soybean. We first analyzed the FPKM (Fragments Per Kilobase of transcript per Million mapped reads) values of PT (phosphate transporter) family genes in soybean roots by using RNA-seq data. Notably, the expression level of *GmPT4* (*Gm10G006700*^24^) was found to be the highest among 14 Pi transporter genes in roots (Fig. 4a). Functional investigation revealed that *GmPT4* was a root-specific expressed gene in soybean (Fig. 4b), and its expression was highly induced by P deficiency in all root tissues, including in root tips, lateral roots, and tap roots (Supplementary Fig. 12). Meanwhile, GmPT4 was localized at the PM of tobacco cells (Supplementary Fig. 11), and was shown to facilitate phosphate transport in yeast (Supplementary Fig. 13). Overexpressing *GmPT4* in hairy roots led to better growth performance and enhanced P uptake in soybean, while knockdown of *GmPT4* resulted in the opposite effect (Supplementary Fig. 14). Furthermore, *GmPT4*-overexpression plants showed significantly higher P-acquisition efficiency and biomass than WT (Fig. 4d–f). Taken together, these results demonstrated that GmPT4 is a PM-targeted Pi transporter responsible for Pi uptake in soybean roots.

To understand the relationship between GmPHF1 and GmPT4 in soybean, we investigated V (Pi tracer) uptake in transgenic hairy roots of soybean. In WT, either overexpressing *GmPHF1* or *GmPT4* significantly increased V uptake, while GmPT4 knockout by Crispr-Cas9 technology or silencing GmPHF1 decreased V uptake (Fig. 4c, upper panel). In contrast, neither overexpressing nor GmPT4 knockout affected V uptake in GmPHF1-RNAi lines (Fig. 4c, upper panel), suggesting that the GmPT4-dependent P uptake in soybean roots was eliminated by silencing *GmPHF1*. Rubidium (Rb, K tracer), which was simultaneously added as an internal control, exhibited consistent uptake levels among various transgenic roots (Fig. 4c, lower panel). In parallel, we generated heritable transgenic plants overexpressing either or both of *GmPHF1* and *GmPT4*, which showed significantly larger P-acquisition efficiency and biomass than WT; no significant differences in P-acquisition efficiency and biomass were observed among these overexpression plants (Fig. 4d–f). These results demonstrate that GmPHF1 modulates P-acquisition efficiency mainly through GmPT4. Further, a membrane yeast two-hybrid assay was carried out and revealed the interaction of GmPHF1 and GmPT4 in vitro (Supplementary Fig. 15), suggesting that GmPHF1 may interact and facilitate GmPT4 exit from ER.

To further verify the effects of GmPHF1 on GmPT4 trafficking, we performed in situ immunostaining of transgenic hairy roots in WT and RNAi lines carrying *ProPT4:gPT4-GFP*. Interestingly, the GmPT4-GFP chimeric protein was specifically targeted to the outer face of the root epidermal cells PM in WT plants, showing a polar localization at the side of the PM facing toward the external rhizosphere (Fig. 4g), further confirming its function in P uptake. In the RNAi line, most of the signal of the GmPT4-GFP protein was observed in ER (Fig. 4g and Supplementary Fig. 16). Nonetheless, a fraction of GmPT4-GFP protein in the RNAi line still displayed polar localization as in the WT. These results indicate that GmPHF1 may facilitate GmPT4’s exit from ER to the PM of root epidermal cells and that the polar localization of GmPT4 is likely independent of GmPHF1.

### Identification of causal variant in *GmPHF1*

**Fig. 5:**
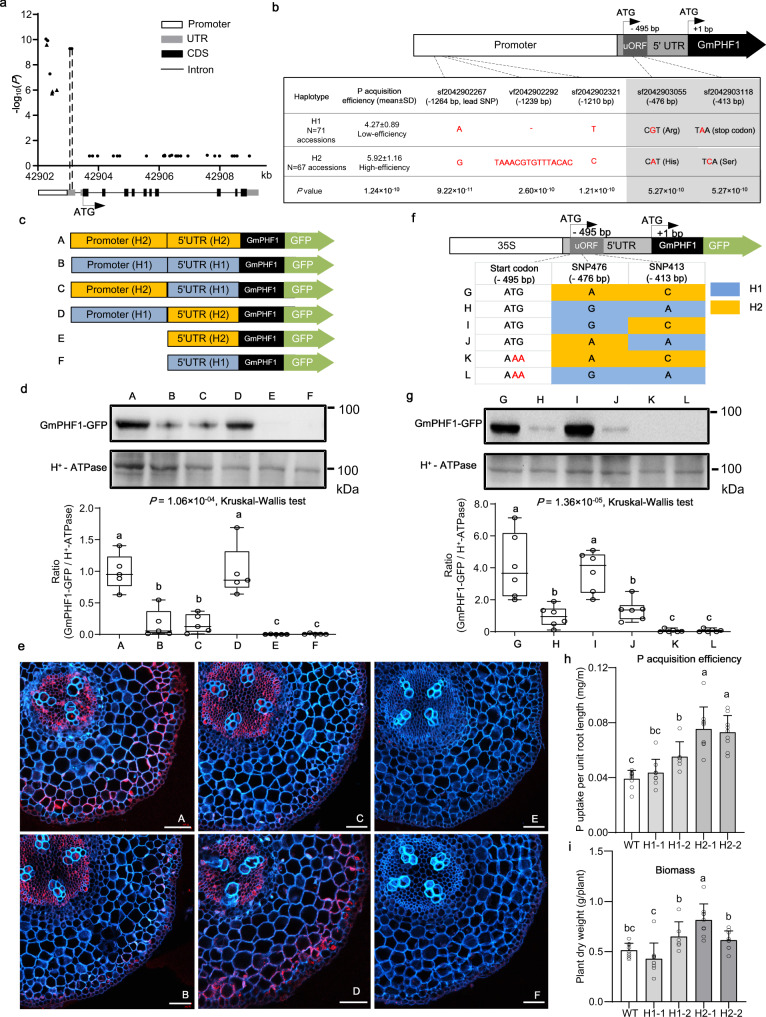
Investigation of the causal variants of *GmPHF1*. **a** *GmPHF1*-based association mapping of P-acquisition efficiency. The dots and triangles represent SNPs and INDELs, respectively. **b** Natural variants between the two haplotypes of GmPHF1. P-acquisition efficiency of two haplotypes were compared by two-sided *t*-test and the *P* value was shown. *P* values of five significant variants in **a** are shown. **c** Recombinant constructs harboring GmPHF1-GFP coding region driven by promoter-5’UTR from different haplotypes. **d** Western blot of GmPHF1-GFP and relative intensity in transgenic hairy roots carrying the constructs in **c**. *n* = 5 biologically independent samples. **e** Immunostaining of GmPHF1-GFP in cross sections of transgenic hairy roots carrying the constructs in **c**. Red fluorescence is the signal from the GFP antibody and Cyan fluorescence is the signal from cell wall. Five independent transgenic lines were investigated and a representative result was shown. Bar = 50 μm. **f** Recombinant constructs (G–J) harboring GmPHF1-GFP coding region driven by 5’UTR of different genotypes of SNPs; construct (K) and (L) harboring H2- and H1–5’UTR with artificial mutation of start codon of uORF (ATG → AAA), respectively. **g** Western blot of GmPHF1-GFP and relative intensity in transgenic hairy roots carrying the constructs in **f**. *n* = 6 biologically independent samples. **d**, **g** The center lines, bounds of box, whiskers, points of boxplot indicate median, lower/upper quartile (25th/75th percentile), minima/maxima, raw data, respectively. *P* values were calculated using Kruskal–Wallis test and different letters indicate a significant difference (*P* < 0.05). H^+^-ATPase is used as an internal control. **h**, **i** Phenotypic parameters including P-acquisition efficiency (**h**) and plant biomass (**i**) of the WT and heritable transgenic lines, which include two independent lines (H1-1, H1-2) harboring H1 promoter + H1–5’UTR: GmPHF1 and two independent lines (H2-1, H2-2) harboring H1 promoter + H2–5’UTR: GmPHF1. Data are presented as mean values + SD. *n* = 9, 9, 6, 9, 9 biologically independent samples, respectively. *P* values are 1.81 × 10^−08^ in **h** and 4.24 × 10^−06^ in **i** based on one-way ANOVA. Different letters indicate significant differences based on Tukey’s multiple-comparison test (*P* < 0.05). Source data underlying panels a, d, g–i are provided as a Source Data file.

*GmPHF1*-based association analysis revealed that significant variants were only enriched in the promoter region and 5’UTR, but not in exon, intron, or 3’UTR regions of *GmPHF1* (Fig. 5a). Based on the significant variants, two major haplotypes (H1 and H2) were identified (Fig. 5b). There were five sequence variants between the two haplotypes, which were significantly associated with P-acquisition efficiency: two SNPs and one INDEL in the promoter region, and two SNPs in the 5’UTR (Fig. 5b). To investigate the causal polymorphism, we first randomly selected five accessions harboring haplotype H1 and another five accessions harboring haplotype H2. We re-sequenced the main ORF (mORF) region of *GmPHF1* in these ten accessions, and found no differences in its amino acid sequence (Supplementary Fig. 17a). Then we compared their expression levels of *GmPHF1* in roots. The real-time RT-PCR results showed that no significant differences in gene expression were observed between the two haplotype groups (Supplementary Fig. 17b). These results indicate that the causal polymorphism is attributed neither to protein sequence nor to gene expression.

Notably, two SNPs within the 5’UTR located at an upstream open reading frame (uORF) (Fig. 5a, b), were in complete LD with each other and had a common frequency (MAF = 0.49). For H1 (low-efficiency haplotype), the SNP sf2042903118 (located at −413 bp of *GmPHF1*, termed “SNP413” hereinafter) introduced a stop codon (T**C**A → T**A**A) and thus shortened the encoding region of the uORF from 39 to 27 amino acids; the SNP sf2042903055 (located at −476 bp of *GmPHF1*, termed “SNP476” hereinafter) replaced a triplet codon for His (C**A**T) by a triplet codon for Arg (C**G**T) (Fig. 5b and Supplementary Fig. 18**)**. Given that uORF may affect the translation of *GmPHF1* transcripts by ribosome stalling^25^, we first quantified the GmPHF1-GFP protein levels in hairy roots driven by different recombinant upstream regions (Fig. 5c). Without the promoter region, the 5’UTR alone failed to initiate *GmPHF1* expression to produce proteins (Fig. 5d). Interestingly, regardless of the promoter region of different haplotypes, the relative protein intensity of GmPHF1 was significantly higher in the hairy roots carrying the H2- than the H1–5’UTR (Fig. 5d). Further immunostaining of these transgenic hairy roots revealed the alteration in tissue distribution of GmPHF1. H2–5’UTR positioned GmPHF1 at stele and outer cortex while H1–5’UTR restricted GmPHF1 only at stele (Fig. 5e), which suggests the uORF’s regulatory activity in a tissue-specific manner and the importance of two SNPs at uORF for the spatial abundance of *GmPHF1* in soybean roots.

To determine the causal SNP, we quantified the GmPHF1-GFP protein levels in hairy roots driven by different recombinant 5’UTRs (Fig. 5f, g). Strikingly, regardless of different haplotypes of 5’UTR, the GmPHF1 failed to be translated when the start codon of uORF was artificially mutated (ATG → AAA) (Fig. 5f, g and Supplementary Fig. 19), indicating the uORF is required for the downstream GmPHF1 translation. Besides, replacing SNP476 (A → G) in H2–5’UTR did not affect GmPHF1 protein levels or distributions. However, replacing SNP413 (C → A) in H2–5’UTR, which truncated one-third of the original full-length uORF, led to remarkable reductions in downstream protein abundance and stele-restricted localization of GmPHF1 (Fig. 5g and Supplementary Fig. 19). These results indicate that the SNP413 is the causal variant, which alters GmPHF1’s abundance in a tissue-specific manner.

Beyond the effect on translation, uORF may trigger nonsense-mediated mRNA decay^26^. To understand whether the uORF’s variant affects steady-state mRNA patterns, a developed technique was used to simultaneously observe the mRNA and protein distribution in situ^27^. We fused tdTomato with an mRNA reporter 3WJ-4 × Bro, which can be used to visualize the promoter-driven tdTomato RNA distribution through DFHBI-1T dye. Notably, the reporter genes driven by the same 35S-promoter but different uORFs, differed in both protein and mRNA patterns (Supplementary Fig. 20). We reason that the uORF’s variant may also affect mRNA stability at post-transcriptional level through nonsense-mediated decay.

To further demonstrate the contribution on P-acquisition efficiency of the uORF, we generated heritable transgenic soybean plants (No. FC010 of H1 haplotype), and found that plants harboring the exogenous H1 promoter + H2 5’UTR: GmPHF1 showed significantly larger P-acquisition efficiency than those harboring exogenous H1 promoter + H1 5’UTR: GmPHF1 (Fig. 5h), although the biomass was not prominently affected (Fig. 5i). We next demonstrated that the improved P acquisition is not due to the small peptides encoded by the uORF, as only introducing promoter with 5’UTR without a coding region did not improve P acquisition (Supplementary Fig. 21).

## Discussion

The low availability of P in soils has led to agricultural practices where farmers use excess amounts of P fertilizers in agricultural systems, which results in a high financial burden for farmers and negative environmental consequences due to P runoff and pollution of surface and groundwater. Genetic approaches to improve crop P acquisition have been worked on for decades but the progress has been limited^8^. In this study, we re-sequenced the whole genomes of 274 accessions in a P-efficiency applied soybean core collection and analyzed its population structure. The close relationship between population structure and geographical distribution seems common as similar results were also found in other soybean populations^13,28^. Considering that the P status in soils is very difficult to simulate in artificial growth media, a field experiment was conducted in order to discover useful loci better suited for crop breeding. The observed large variation in P acquisition (Fig. 2a–d) in the field study of our soybean association panel inspired us to conduct GWAS to reveal natural genetic variation in P-acquisition efficiency. Though it has a relatively smaller sample size, GWAS using the South soybean subpopulation may have higher mapping power than in the whole population (Supplementary Figs. 5 and 6), which could be due to: (1) the genetic heterogeneity across subpopulations may result in relatively lower mapping power in the whole population, (2) the field experiment was performed in South China, where the accessions in the South subpopulation were originally located. Hence the phenotypic data of locally-adapted accessions from the South subpopulation could not be biased by environmental filtering and thus could better reflect the genetic effect of QTLs. Therefore, if trade-offs between diversity and adaptation of a natural population are not fully considered, large sample size does not necessarily provide higher mapping power in GWAS.

Consistent with the previous reports^7,11^, the correlation between P uptake and root length was observed in this study (Fig. 2c). Indeed, increasing root length is an important strategy to absorption of relatively immobile P in soils as it enlarges the soil contact area for P acquisition. However, large variation in P uptake which failed to be explained by root length (Fig. 2c), as well as variations in P-acquisition efficiency (P uptake per unit root length) were observed in the study (Fig. 2d), suggesting that there exist genetic loci which contribute to P acquisition independently of root growth traits. Interestingly, after adjusting for root length, some association signals were attenuated, and the *P* value inflation was better controlled (Supplementary Fig. 6). The loci with attenuated association signals may be spurious associations due to environmental noise or true QTLs which control the studied trait by regulating the root length (Supplementary Fig. 6). Notably, this adjustment increased mapping power to detect *CPU1* in the study (Supplementary Fig. 6). As a complex trait is governed by a number of minor loci, GWAS usually does not have sufficient statistical power to detect these loci and distinguish them from background signals. As an alternative solution to this dilemma, adjustment for a heritable covariate removes the variance explained by the added covariate out of the total phenotypic variance, and hence increases the ratio of variance contributed by a true covariate-independent QTL, resulting in improved statistical power^29^.

As a common mechanism observed in both dicots and monocots, PT family member-mediated Pi transport at the PM^30–34^, plays a pivotal role in plant P homeostasis and has exhibited potential for improving crop P acquisition^20,21,35^. In our study, we found a key component associated with P-acquisition efficiency in soybean, which is not a Pi transporter but rather a regulator that facilitates the ER exit of an important root Pi uptake transporter to the PM of root epidermal cells (Figs. 3, 4). Since direct genetic association between root P efficiency and Pi transporters was not found in rice natural population either^36^, it is suggested that the natural genetic variation of P-acquisition efficiency is more likely related to Pi transport regulators rather than Pi transporter activity in plants.

SEC12 is a guanine nucleotide exchange factor (GEF), which activates a small GTPase Sar1 to initiate COPII-coated vesicle assembly for protein and lipid transport from the endoplasmic reticulum (ER) to the Golgi. *CPU1*’s causal gene, *GmPHF1*, like its homologs in *Arabidopsis* and rice, is structurally related to SEC12, but shared only three out of ten conserved amino acids among SEC12 homologs in plants, animals, and yeasts. In *Arabidopsis*, AtSEC12 could rescue the growth defect of the *sec12*-mutant yeast but AtPHF1 could not^20^. These results suggest functional divergence between PHF1 and SEC12. The interaction between SEC12 and Sar1 has been studied via crystal structure analysis and structure-guided site-directed mutagenesis. The K loop of SEC12 (residues 29-40, GGGG----GΦ-N, where Φ represents a hydrophobic amino acid) makes direct contact with Sar1, of which the Asn (N) at residue 40 is critically important for GEF activity; the Sar1-Sec12 interaction interface involves 25 residues of Sar1, most of which are in the switch II region (residues 76-92). GmPHF1 lacks the conserved residues for the K loop of SEC12 and PTs lack similarity in both protein sequence and secondary structure with Sar1, which suggests SEC12-Sar1 interaction model may not be applicable for the PHF1-PT interaction. Although it is known that the PHF1-PT interaction is repressed by PT’s phosphorylation^22^, fully understanding the interacting mechanism still requires further studies of crystal structure or structure-guided site-directed mutagenesis. Besides, the location of GmPHF1 in roots (at stele and outer cortex) (Fig. 5e) is wider than GmPT4 (only at epidermis) (Fig. 4g), suggesting the existence of other targeting proteins of GmPHF1 at stele or cortex.

To date, almost all published natural variations contributing to phenotypic diversity in plants are involved in either gene expression controlled by promoter polymorphisms^37^, or protein activity caused by coding region differences^38^. However, as a key step for protein functioning, natural variation of translational regulation from mRNA to protein is often overlooked in plants^25^. As a regulatory cis-element controlling translational levels of eukaryotic genes, uORF in the 5’UTR usually represses the translation of the downstream mORF via ribosome stalling and nonsense-mediated decay^39^, and few examples of enhancer uORF have been previously reported^40^. The uORF discovered in our study, was demonstrated to be indispensable for the downstream protein translation. Both knockout of this uORF by artificially mutating its start codon and truncating this uORF’s length by the naturally occurring SNP413, remarkably altered GmPHF1’s abundance in a tissue-specific manner in roots (Fig. 5f, g and Supplementary Fig. 19). The causal SNP413 could be developed into a molecular marker for breeding of high-P-efficiency soybean in the future. Since enhancer uORFs in plants are rarely reported and the underlying mechanisms are not well-studied, our study provides a good model for a better understanding the mechanism of uORF in enhancing downstream gene translation.

The regulations at the transcriptional, post-transcriptional, and translational levels sequentially determine the final protein abundance of GmPHF1, and the difference in GmPHF1 abundance/patterns between the H1- and H2-allele could be possibly caused by these three processes. Even though the variant in 5’UTR might affect the binding of a transcription factor^41^, the contribution of transcriptional regulation could be minor in our study because the causal SNP changes the length of a uORF, which often functions at the post-transcriptional and translational levels^42^. uORF is known to play instrumental roles in translation control of the downstream main ORF in a broad spectrum of eukaryotes^43^. Furthermore, when a uORF is translated, a long 3’UTR downstream of the uORF may activate nonsense-mediated decay to affect mRNA stability and thus steady-state mRNA levels^26^. In this study, we discovered the impact of uORF on both steady-state mRNA level and protein abundance (Supplementary Fig. 20), suggesting the possibility of uORF-triggered mRNA decay of *GmPHF1*. In parallel, consistent with previous reports^44,45^, we found the tissue-specific regulatory activity of uORF (Fig. 5e and Supplementary Fig. 19), which suggests that selective expression of uORFs could be a key regulatory factor for the downstream genes.

In conclusion, our study revealed a locus *CPU1* associated with P acquisition using GWAS in soybean. The causal gene, *GmPHF1*, encodes a Pi transporter protein translocation facilitator that enables the localization of a Pi transporter GmPT4 at the PM of root epidermis for better P uptake. A naturally occurring variant in uORF led to different GmPHF1 abundance, and thereby contributed to P-acquisition diversity in soybean. Our study has helped broaden the scope for the natural genetic variation underlying phenotypic diversity of plants, and provided a potential genetic resource for improving P acquisition in soybean.

## Methods

### Phenotyping a soybean natural population

The P-efficiency applied core collection used in the study was constructed using a GIS-assisted approach based on soil types and soil P status, agricultural divisions and distributions of soybean, and their agronomic traits^15,16^. The field experiment was conducted during the summer sowing season in Boluo, Guangdong, P.R. China (east longitude 113°50′, north latitude 23°07′). The soil chemical properties were analyzed as follows: pH value: 2.5:1 (water/soil); organic matter using K_2_Cr_2_O_7_-H_2_SO_4_ digestion; total N content using Kjedahl method; total P content using H_2_SO_4_-HClO_4_ digestion; total K content using NaOH fusion; available N content using alkaline diffusion; available P content using Bray II method; available K content using 1 mol/L neutral NH_4_OAc extraction (Supplementary Table 4).

For the field experiment, a randomized complete block design was followed with four replications. Each plot had 1.5-m^2^ area and planting density was 30 cm × 10 cm. For the first harvest, one soybean plant of each accession for each replication was harvested at the seedling stage (1 month after sowing) to measure plant dry weight, P content and root length. According to their maturity time, plants were harvested the second time to measure yield. Averaged values of the four replications for the four traits were calculated.

P content was analyzed using colorimetric methods^46^. Harvested shoots were dried at 105 °C in an oven for 30 min and at 75 °C to constant weight to weigh the dry weight. To harvest the complete root system of a plant, a soil square block of 40 cm × 40 cm with plant base as center was dug down to the end of taproot. The harvested roots were brought to the lab and were cleaned with water. Root length was quantified using computer image analysis software (WinRhizo Pro, Régent Instruments, Québec, Canada) based on the root images acquired by a scanner with a blue board as a background.

### Analysis of phenotypic data

Kruskal–Wallis test, two-sided *t*-test, Tukey’s multiple-comparison test, and Pearson correlation analyses were conducted using IBM SPSS version 19 (IBM, Armonk, USA).

### Re-sequencing and variant calling

Leaf tissues of each accession were collected for genomic DNA extraction. Sequencing libraries with insert size of ~350 bp were constructed for each accession following Illumina’s standard protocol. Paired-end reads were generated and sequenced using the Illumina NovaSeq PE150 platform, producing ~13.5 billion reads and ~2 Tb (terabases) for 274 accessions. All the DNA extraction, library construction, and sequencing were performed by Novogene Bioinformatics Technology Co., Ltd, China. The Illumina sequencing data could be downloaded from National Center for Biotechnology Information Sequence Read Archive under accession number PRJNA633739.

After removing low-quality reads by fastp v0.19.4^47^, the remaining reads were aligned to the soybean reference genome (Wm82.v2.0.40 from EnsemblPlants, http://plants.ensembl.org/info/website/ftp/index.html) using bwa v0.7.17^48^. Unmapped reads, secondary alignments, and duplicate reads were removed using SAMtools v1.9^49^. Alignment was re-performed in the region around INDEL sites using GATK v3.8^50^. Quality control for BAM alignments was performed by using Qualimap v.2.2.1^51^. Joint-genotyping of 274 accessions was conducted using GATK for SNP and INDEL calling. SNPs with “DP < 10” or “QD < 2.0” or “FS > 60.0” or “SOR > 4.0” or “MQ < 40.0” or “MQRankSum < −3.0” or “ReadPosRankSum < −3.0” or “QUAL < 30” were removed using GATK. The SNPs were further filtered using PyVCF (https://pyvcf.readthedocs.io/en/latest/) with the parameters “avg-dps --avg-depth-per-sample 3 eb --eblr −10”. For the homozygous genotype matrix of bi-allelic SNPs, genotype imputation was performed to infer the missing genotypes using Beagle v4.1^52,53^. The SNPs with minor allele frequency (MAF) less than 0.01 were removed and the effects of 6,300,394 high-quality SNPs were annotated using snpEff v4.3^54^. The low-quality INDELs with “DP < 10” or “QD < 2.0” or “FS > 200.0” or “SOR > 10.0” or “InbreedingCoeff < −0.8” or “ReadPosRankSum < −20.0” or “QUAL < 30” were removed and the annotation of INDELs’ effects was also conducted using snpEff^54^. Each SNP or INDEL was labeled with a unique identifier, of which the first letter “s” and “v” represent SNP and INDEL, respectively, and the second letter “f” represents the soybean reference genome Wm82.v2.0.40 from EnsemblPlants, and the number is comprised of chromosome ID and chromosome coordinate. For example, sf2042902267 indicate a SNP at the 42902267 bp of chromosome 20.

### Characterization of the core collection based on genotype data

To understand genetic structure of 274 soybean accessions in the core collection, based on genotypes of genome-wide SNPs, PCA (Principal Component Analysis) was performed using GCTA (Genome-wide Complex Trait Analysis) v1.24.4^55^, and a neighbor-joining tree was constructed using MEGA7-CC (command-line version of MEGA (Molecular Evolutionary Genetics Analysis))^56^ and visualized using R package “ggtree”^57^. Estimation of ancestry for the core collection and calculation of subpopulation component values were performed by using ADMIXTURE v1.3.0^58^. Accessions were assigned into subpopulations based on their maximum subpopulation component values. An accession with the difference between the maximum subpopulation component value and the second-largest subpopulation component value less than 0.4, was not assigned into any subpopulation and was labeled as “Intermediate”^59^. Genome-wide population differentiation analysis was conducted to identify greatly divergent genomic regions using VCFtools v0.1.16^60^ with the parameters “--fst-window-size 100000 --fst-window-step 100000”. Adjacent 100-kb-windows with *F*_ST_ value ≧0.65 (top 5% *F*_ST_) separated by no more than 2-Mb distance, were merged into a divergent genomic region; *F*_ST_ value of the merged divergent genomic region was the maximum of *F*_ST_ values of 100-kb windows within the merged region. Genome-wide and chromosome-wide LD-decay distances were estimated and LD-decay plots were made by using a Perl tool, PopLDdecay v3.40^61^, with default parameters. Circos plots were made using shinyCircos^62^.

### Genome-wide association study

Genome-wide association studies were performed in the whole population and the South subpopulation, respectively. Both the SNPs with a minor allele frequency (MAF) less than 0.05 were removed from association analysis. A total of 4,618,874 and 3,757,697 SNPs were used for GWAS in the whole population and the South subpopulation, respectively. To control false-positive associations due to multiple levels of relatedness, we performed the Linear Mixed Model (LMM) fitting identity-by-state kinship matrix (K) as a random effect using the FaST-LMM v2.07^63^. The identity-by-state kinship matrix (K) was calculated using the function “--ibs-matrix” of Plink v1.90^64^. The genome-wide significance was determined by the Bonferroni correction (0.05/Ne), in which Ne was the effective number of independent SNPs calculated by the GEC v0.2^65^. The significance thresholds were 1.21 × 10^−07^ and 1.84 × 10^−07^ for the whole population and the South subpopulation, respectively. Independent lead SNPs or loci were obtained using the function “--clump” of Plink^64^ to reduce redundant significant SNPs with the LD statistic (*r*^2^) ≧ 0.30. To perform GWAS with adjustment for root length, GWAS were performed with adding root length as a covariate by using the function of “-covar” of FaST-LMM^63^. Haplotypes were determined based on the genotypes of significant SNPs.

### Plant material and growth conditions

The soybean (*Glycine max L*.) wild-type (YC04-5, harboring *GmPHF1* high-efficiency haplotype (H2)) and RNAi lines of *GmPHF1* were germinated and grown in vermiculite. Seedlings were watered with the nutrient solution every day^66^, and grown in a growth chamber with a 13 h/26 °C day and 11 h/24 °C night regime, a daytime light intensity of 400 μmol photons m^−2^ s^−1^, and relative humidity of 65%.

To generate the RNAi lines, a 147-bp fragment of the *GmPHF1* coding sequence was amplified using the primers F1/R1 (Supplementary Data 5) and then cloned into *Asc*I and *Swa*I sites, or *Sma*I and *Bam*HI sites for the sense and antisense orientation of the *pFGC5941* (GenBank Accession No. AY310901), respectively. To construct overexpression lines, the entire ORF of *GmPHF1* and *GmPT4* was amplified by PCR and respectively inserted into the *Asc*I and *Bam*HI sites of *pFGC5941* vector driven by *CaMV35S* promoter according to the protocol of ClonExpress^®^ II One Step Cloning Kit (Vazyme). Primer pairs used for amplification were F21/R21 for *GmPHF1*; F22/R22 for *GmPT4*. Transgenic seedlings were then obtained through *Agrobacterium tumefaciens* (EHA105)-mediated transformation^67^.

At the seedling stage (18-d-old), roots were scanned (WinRHIZO LA2400, Regent Instruments, Québec, Canada), and total root length was calculated in WinRHIZO software (Regent Instruments, Québec, Canada). Shoots and roots were separately harvested and dried at 65 °C for 2 days and then weighed. Samples were digested with 3 mL concentrated HNO_3_. Elemental concentration was determined by inductively coupled plasma-mass spectrometry (ICP-MS, Agilent 7900, Agilent Technologies, SantaClara, CA, USA). At the maturity stage (100-d-old), pods were detached from stem and pod number was counted for each plant.

### Yeast two-hybrid assay

The interactions between GmPHF1 and GmPT4 proteins were tested through DUALmembrane kit 3 (Dualsystems Biotech AG, Switzerland) as GmPHF1 and GmPT4 are membrane proteins. The ORF of *GmPT4* was amplified using the primers F2/R2, and ligated to a signal sequence derived from the *SUC2* (invertase) gene of *Saccharomyces cerevisiae* through overlapping PCR using the primers F3 (signal sequence of *SUC2*) and F4/R2 (primers used to amplify the *SUC2-GmPT4* products). The products were then cloned into *Nco*I and *Hind*III sites of the bait vector pBT3-C to create GmPT4-Cub (C-terminal fragment of ubiquitin). The ORF of *GmPHF1* was amplified using the primers F5/R5 and cloned into *BamH*I and *EcoR*I sites of the prey vector pPR3-N to create Nub-GmPHF1 (N-terminal fragment of ubiquitin). Primers were listed in Supplementary Data 5. GmPT4-Cub and Nub-GmPHF1 were co-transformed into the yeast strain NMY51 (*his3, trp1, leu2, ade2, LYS2::HIS3, ura3::lacZ, ade2::ADE2, GAL4*) using the S.c.easy Comp Transformation Kit (Invitrogen, CA, USA) according to the manufacturer’s protocols. The construct pAI-Alg5 expressing a fusion of the yeast ER protein Alg5 to the wild-type Nub portion (NubI) of yeast ubiquitin was used as the positive control prey. pDL2-Alg5 expressing a fusion of the same protein to the mutated Nub portion (NubG) was used as the negative control prey.

Yeast transformants were selected on synthetic defined medium without leucine and tryptophan (SD-Leu-Trp). The positive transformants were then spotted onto SD-Leu-Trp medium with or without X-gal for β-galactosidase assay. Plates were incubated for 48 h at 30 °C before being photographed.

### Transcriptomic and gene expression analysis

Soybean seedlings were cultured for 3 weeks before sampling the roots for RNA-seq analysis. The samples were quickly frozen by liquid N and provided to Novogene for RNA sequencing analysis using an Illumina HiSeq 2500 (Novogene Biotechnology, Guangzhou, China).

To investigate the expression of *GmPHF1* in different haplotypes, soybean genotypes with low-efficiency haplotype (H1) and high-efficiency haplotype (H2) were germinated and grown in vermiculite. After being watered with the nutrient solution for 12 d, the roots from soybean seedlings were harvested for RNA extraction. To determine *GmPHF1* expression in RNAi lines, the seeds of wild-type (WT, YC04-5) and three RNAi lines of *GmPHF1* were germinated and grown in vermiculite for 18 days. The true leaves were sampled for RNA isolation. To investigate *GmPT4* expression, WT soybean seeds were germinated on moistened paper towels for 2 d, and then transplanted to nutrient solution for 2 d. The seedlings were then treated with low P (5 μM) or Normal P (250 μM) for 7 d. Root tip, lateral root, and taproot were separately harvested for RNA isolation. To investigate spatial expression of *GmPT4*, root, stem, leaf, and flower samples of soybean seedlings were harvested at 30 d and pod at 40 d.

Total RNA was extracted from the frozen samples using TransZol Up Plus RNA Kit (TransGen, Beijing, China) following the manufacturer’s protocols. In all, 500 ng of extracted RNA was used for first-strand cDNA synthesis using a TransScript One-Step gDNA Removal and cDNA Synthesis SuperMix Kit (TransGen, Beijing, China). Relative gene expression levels were determined by qRT-PCR using TransStart Top Green qPCR Super Mix (TransGen, Beijing, China). The housekeeping gene *EF-1a* was used as an internal control. Primers for real-time RT-PCR were F6/R6 for *GmPHF1*, F20/R20 for *GmPT4*, and F7/R7 for *EF-1α* (Supplementary Data 5). Normalized relative expression was calculated by the ΔΔCt method.

### Construction of recombinant constructs for *GmPHF1*

To explore the effects of natural variation in 5’UTR on the translation of *GmPHF1*, promoter region and 5’UTR from different haplotypes were reassembled. Since haplotype H1 and H2 shared the same main ORF, the main ORF of *GmPHF1* was amplified using the primers F10/R10 and cloned into *EcoR*I and *Asc*I sites of the *pFGC5941-p35S-GFP* vector to create the *GmPHF1-GFP*. The promoter region and its 5’UTR from haplotype H1 and H2 were amplified using the primers F11/R11 individually and cloned into the *Eco*RI site of *GmPHF1-GFP* to create *H1*_*promoter*_ + *H1*_*5’UTR*_*:GmPHF1-GFP* and *H2*_*promoter*_ + *H2*_*5’UTR*_*:GmPHF1-GFP*. The promoter region of H1 and H2 were amplified using the primers F11/R12, while the 5’UTR of H1 and H2 were amplified using the primers F12/R11 individually (Supplementary Data 5). *H1*_*promoter*_ + *H2*_*5’UTR*_ and *H2*_*promoter*_ + *H1*_*5’UTR*_ were separately ligated by overlapping PCR using the primers F11/R11. The PCR products were cloned into the *EcoR*I site of GmPHF1-GFP to create *H1*_*promoter*_ + *H2*_*5’UTR*_*:GmPHF1-GFP* and *H2*_*promoter*_ + *H1*_*5’UTR*_*:GmPHF1-GFP*. Finally, the 5’UTR of H1 and H2 were amplified separately using the primers F13/R11 and cloned into the *EcoR*I site of *GmPHF1-GFP* to create *H1*_*5’UTR*_*:GmPHF1-GFP* and *H2*_*5’UTR*_*:GmPHF1-GFP*.

To determine the causal SNP at the uORF, we quantified the relative intensity of GmPHF1-GFP protein in hairy roots transformed with different recombinant vectors. The main ORF of GmPHF1 was amplified using the primers F14/R10 and cloned into the *Asc*I site of the *pFGC5941-p35S-GFP* vector to create the *p35S:GmPHF1-GFP*. The native 5’UTR of H1 and H2 were amplified separately using the primers F15/R15 individually. Two SNPs within the 5’UTR were reassembled. 5’UTR containing the first SNP from H1 and the second SNP from H2 were amplified by overlapping PCR using the primers F16/F17/R15 (F17 contained the first SNP from H1). 5’UTR with the first SNP from H2 and the second SNP from H1 were amplified by overlapping PCR using the primers F15/F18/R18/R15 (F18 and R18 contained the second SNP from H1 and were reverse complementary). 5’UTR with the mutated form of uORF (ATG start codon changed to AAA) from H1 and H2 were amplified using the primers F19/R15 individually (Supplementary Data 5). All the six PCR products were cloned into the *Asc*I site of *p35S:GmPHF1-GFP* for further causal SNP determination. These constructs were transformed into hairy root for western blot analysis and immunostaining assay.

### Western blot analysis

For western blot analysis, the transgenic hairy roots were harvested and immediately ground into powder in liquid N. Sample was loaded equally onto an SDS-PAGE gel, and then blotted to a polyvinylidene fluoride membrane (Immobilon-P, Millipore, MA, USA). The membrane was then probed with anti-GFP antibody (1:1000; TransGen, Beijing, China) or anti H^+^-ATPase (1:2000; Agrisera, Vännäs, Sweden) overnight and their corresponding horseradish peroxidase (HRP)-conjugated second antibodies (anti-mouse IgG for GFP (1:5000; TransGen, Beijing, China); anti-rabbit IgG for ATPase (1:5000; Biosharp, Hefei, China)) for 1 h. HRP signals were detected using the SuperSignal West Dura Trial Kit (Thermo Scientific, MA, USA) with an Amersham Imager 600 System (GE Healthcare Bio-Sciences AB, Uppsala, Sweden).

### Immunostaining assay

Immunostaining was performed according to the methods in ref. ^68^. Briefly, transgenic hairy-root segments (10 mm from root tips) were kept in fixative solution [4% (w/v) paraformaldehyde, 60 mmol/L sucrose, and 50 mmol/L sodium cacodylate (pH 7.4)] for 2 h prior to slicing 100-μm-thick sections with a microtome (LEICA RM2235, Leica Microsystems GmbH, Wetzlar, Germany). Sections were incubated with 0.3% (v/v) Triton X-100 for 2 h, followed by the anti-GFP (1:1000; Thermo Fisher Scientific, Somerset, NJ, USA) primary antibody incubation overnight and the secondary antibodies (Alexa Fluor 555 goat anti-rabbit IgG; 1:2000; Molecular Probes, Eugene, OR, USA) for 2 h. Fluorescence was observed with a confocal scanning microscope (LSM880, Carl Zeiss, Oberkochen, Germany).

### Subcellular localization of GmPHF1 and GmPT4

To investigate the subcellular localization of GmPHF1 and GmPT4 in tobacco leaves, the ORF of *GmPHF1* was amplified by PCR using the specific primers F14/R14 (Supplementary Data 5), and inserted into the *Sal*I and *BamH*I sites of pBEGFP to generate *p35S:GmPHF1-GFP*. The ORF of *GmPT4* was amplified by PCR using the specific primers F15/R15 (Supplementary Data 5), and cloned into the pDONR207 entry vector, then sub-cloned into the pMDC43 gateway binary vector using Gateway LR Clonase II Enzyme Mix (Invitrogen, Carlsbad, CA, USA) to generate *p35S:GmPT4-GFP*. These recombinant vectors were co-expressed with *CD3-1008* (PM marker) or *CD3-960* (ER marker) in tobacco leaves using *Agrobacterium*-mediated transformation^23^.

To investigate subcellular localization of GmPT4 in soybean roots, the 2384 bp of the *GmPT4* promoter was amplified using the primers F8/R8, and then cloned into *EcoR*I and *Asc*I sites of the *pFGC5941-p35S-GFP* vector^68^ using the ClonExpress II One Step Cloning Kit (Vazyme, Nanjing, China). Subsequently, the genomic DNA of *GmPT4* was amplified using the primers F9/R9 and cloned into the *Asc*I site of the recombinant vector to create *ProPT4:gPT4-GFP*. The *ProPT4:gPT4-GFP* and *p35S:GmPHF1-GFP* constructs were individually transformed into *Agrobacterium rhizogenes* strain K599 for hairy-root transformation^68^ and subsequent immunostaining.

### LA-ICP-MS analysis

Soybean seedlings (5-d-old) were treated with 1 μM V for 6 h, and the root segments (10 mm from root tips) were quickly sliced into 100-μm-thick sections with a microtome on ice, and displayed in a sample freezer at −20 °C (CryoCell, Elemental Scientific) to stabilize sample shape and prevent ion flow. Samples were analyzed using a LA unit (NWR213; New Wave Research) equipped with an Nd:YAG solid-state laser source operating at 213 nm with the following settings: energy: 35% of maximum energy; scan speed: 15 µm s^−1^; repetition rate: 5 Hz; spot size: 10 µm. Element signals were obtained using an Agilent Technologies 7900 ICP-MS instrument operated in helium mode (4.8 mL/min). The isotopes were analyzed using an integration time of C^13^ (0.01 s), P^31^ (0.02 s), and V^51^ (0.1 s). All element signals were normalized to C^13^ and converted to element image using iolite 4 software (http://iolite-software.com/).

### V and Rb uptake assay

To construct *p35S:GmPHF1* and *p35S:GmPT4*, the entire ORF of *GmPHF1* and *GmPT4* was amplified by PCR and respectively inserted into the *Asc*I and *Bam*HI sites of *pFGC5941* vector driven by *CaMV35S* promoter according to the protocol of ClonExpress^®^ II One Step Cloning Kit (Vazyme). Primer pairs used for amplification were F21/R21 for *GmPHF1*; F22/R22 for *GmPT4*. To construct *Crispr-Cas9-GmPT4* vector, the target site primers (F23/R23) was designed and introduced into the pGEL201 vector^69^ using *Bsa*1 and T4 DNA ligase. *p35S:GmPHF1*, *p35S:GmPT4*, *Cas9-GmPT4* constructs were introduced into hairy root of WT (YC04-5), and *p35S:GmPT4*, *Cas9-GmPT4* constructs were introduced into hairy root of GmPHF1-RNAi lines. The transgenic hairy roots were treated with 1 μM V and 1 μM Rb for 12 h, and then dried and digested for elemental determination by ICP-MS. For *Cas9-GmPT4* transgenic hairy roots, those identified as gene editing on *GmPT4* were used for analysis.

### Transport activity assay of GmPT4 in yeast

The ORF of *GmPT4* was amplified using the primers F16/R16 (Supplementary Data 5), and cloned into *Eco*RI and *Not*I sites of the expression vector YP112^70^ to generate GmPT4-YP112. YP112 (negative control) and GmPT4-YP112 were individually transformed into the yeast Pi uptake-defective mutant *MB192*^71^. Yeast transformants were selected on synthetic defined medium without tryptophan (SD-Trp), and spotted onto yeast nitrogen base (YNB) plates containing 50 μM KH_2_PO_4_ at 30 °C for 3 d^72^.

### Phenotypic analysis of GmPT4 knockdown and overexpression lines

To generate the RNAi construct, a 400-bp fragment *GmPT4* ORF was amplified using the primers F17/R17 (Supplementary Data 5), and inserted into the *Asc*I and *Swa*I sites of *pFGC5941* vector for the sense orientation, and then inserted into the *Sma*I and *Xba*I sites for the antisense orientation. To generate overexpression construct, the ORF of *GmPT4* was amplified using the primers F18/R18 (Supplementary Data 5), and cloned into *Xba*I and *Sal*I sites of pTF101.1 s. Recombinant plasmids were introduced into *Agrobacterium tumefaciens* strain K599, and transformed into soybean by hairy-root transformation methods as described in ref. ^68^. P concentration was determined by ICP-MS.

### Genetic validation of causal variants

To validate the effects of natural variation in 5’UTR on the P-acquisition efficiency, H1 promoter region and 5’UTR from different haplotypes were reassembled. The *H1*_*promoter*_ + *H1*_*5’UTR*_ fragment and *H1*_*promoter*_ + *H2*_*5’UTR*_ fragment were amplified from the constructed vectors *H1*_*promoter*_ + *H1*_*5’UTR*_*:GmPHF1-GFP* (construct B in Fig. 5c) and *H1*_*promoter*_ + *H2*_*5’UTR*_*:GmPHF1-GFP* (construct D in Fig. 5c) respectively using primers F27/R27, and then cloned into *EcoR*I and *BamH*I sites of the *pFGC5941-p35S-GFP* vector to create the *pFGC5941-H1*_*promoter*_ + *H1*_*5’UTR*_ and *pFGC5941-H1*_*promoter*_ + *H2*_*5’UTR*_. The genomic DNA of *GmPHF1* was amplified using the primers F28/R28, and cloned into the *BamH*I site to create *pFGC5941-H1*_*promoter*_ + *H1*_*5’UTR*_-*GmPHF1* and *pFGC5941-H1*_*promoter*_ + *H2*_*5’UTR*_-*GmPHF1* individually. These vectors were introduced into soybean by either Agrobacterium-mediated heritable transformation or hairy-root transformation.

### In situ hybridization of mRNA and protein

A developed technique^27^ was utilized to simultaneously visualize mRNA and protein distribution in soybean roots. Briefly, the tdTomato was amplified using the primers F24/R24 and the 3WJ-4×Bro was amplified using the primers F25/R25, and then the recombinant tdTomato-3WJ-4×Bro fragment was cloned into the *Asc*I and *Bam*HI sites of the *pFGC5941-p35S* vector to create the *p35S: tdTomato-3WJ-4×Bro*. The 5’UTRs from construct H and I (Fig. 5f) were amplified individually using the primers F26/R26, and then cloned into the *Asc*I site of *p35S: tdTomato-3WJ-4×Bro* to create the *p35S: H1 5’UTR-tdTomato-3WJ-4×Bro* and *p35S: H2 5’UTR-tdTomato-3WJ-4×Bro*. The transgenic hairy roots carrying these constructs were infiltrated by 100 μM DFHBI-1T solution (LuceRNA^TM^) for 12 h. Subsequently, root segments (10 mm from root tips) were quickly harvested and sliced into 100-μm-thick sections with a microtome (Leica RM2235; Leica Microsystems GmbH, Wetzlar, Germany) under RNase-free and 0 °C conditions. The DFHBI-1T signals were visualized and photographed by confocal microscopy (LSM880; Carl Zeiss) using a 488 nm argon laser for excitation and 525 nm for emission.

### Reporting summary

Further information on research design is available in the Nature Research Reporting Summary linked to this article.

## Supplementary information

Supplementary Information

Description of Additional Supplementary Files

Supplementary Data 1

Supplementary Data 2

Supplementary Data 3

Supplementary Data 4

Supplementary Data 5

Reporting Summary

## Data Availability

The Illumina sequencing data generated in this study have been deposited in NCBI (National Center for Biotechnology Information) under SRA (Sequence Read Archive) accession number SRR11929594 - SRR11929867 of PRJNA633739. Phytozome database [https://phytozome.jgi.doe.gov/] was used to retrieve the genes’ expression levels and annotations. Soybean Expression Atlas database [https://venanciogroup.uenf.br/cgi-bin/gmax_atlas/index.cgi] was used to retrieve genes’ expression levels of multiple tissues. All data are available in the manuscript or the Supplementary Materials. Source data are provided with this paper.

## Source data

Source Data

## References

[1] Marschner, P. *Marschner’s Mineral Nutrition of Higher Plants* (Academic Press, 2012).

[2] Wang X, Shen J, Liao H (2010). Acquisition or utilization, which is more critical for enhancing phosphorus efficiency in modern crops?. Plant Sci..

[3] Cong WF, Suriyagoda LDB, Lambers H (2020). Tightening the phosphorus cycle through phosphorus-efficient crop genotypes. Trends Plant Sci..

[4] Gutierrez-Alanis D, Ojeda-Rivera JO, Yong-Villalobos L, Cardenas-Torres L, Herrera-Estrella L (2018). Adaptation to phosphate scarcity: tips from Arabidopsis roots. Trends Plant Sci..

[5] Gu MA, Chen AQ, Sun SB, Xu GH (2016). Complex regulation of plant phosphate transporters and the gap between molecular mechanisms and practical application: what is missing?. Mol. Plant.

[6] Kuang RB, Chan KH, Yeung E, Lim BL (2009). Molecular and biochemical characterization of AtPAP15, a purple acid phosphatase with phytase activity, in Arabidopsis. Plant Physiol..

[7] Liang Q, Cheng X, Mei M, Yan X, Liao H (2010). QTL analysis of root traits as related to phosphorus efficiency in soybean. Ann. Bot..

[8] Tian J, Wang XR, Tong YP, Chen XP, Liao H (2012). Bioengineering and management for efficient phosphorus utilization in crops and pastures. Curr. Opin. Biotechnol..

[9] Wissuwa M, Yano M, Ae N (1998). Mapping of QTLs for phosphorus-deficiency tolerance in rice (*Oryza sativa* L.). Theor. Appl. Genet..

[10] Wissuwa M, Wegner J, Ae N, Yano M (2002). Substitution mapping of Pup1: a major QTL increasing phosphorus uptake of rice from a phosphorus-deficient soil. Theor. Appl. Genet..

[11] Gamuyao R (2012). The protein kinase Pstol1 from traditional rice confers tolerance of phosphorus deficiency. Nature.

[12] Hufnagel B (2014). Duplicate and conquer: multiple homologs of PHOSPHORUS-STARVATION TOLERANCE1 enhance phosphorus acquisition and sorghum performance on low-phosphorus soils. Plant Physiol..

[13] Fang, C. et al. Genome-wide association studies dissect the genetic networks underlying agronomical traits in soybean. *Genome Biol*. **18**, 1–14 (2017). 10.1186/s13059-017-1289-9 PMC5571659 28838319

[14] Schmutz J (2010). Genome sequence of the palaeopolyploid soybean. Nature.

[15] Zhao J (2004). Characterization of root architecture in an applied core collection for phosphorus efficiency of soybean germplasm. Chin. Sci. Bull..

[16] Li Q (2014). Environmental controls on cultivated soybean phenotypic traits across China. Agr. Ecosyst. Environ..

[17] Zabala G, Vodkin LO (2007). A rearrangement resulting in small tandem repeats in the F3 ‘ 5 ‘ H gene of white flower genotypes is associated with the soybean W1 locus. Crop Sci..

[18] Li Y, Huang Y, Bergelson J, Nordborg M, Borevitz JO (2010). Association mapping of local climate-sensitive quantitative trait loci in Arabidopsis thaliana. Proc. Natl Acad. Sci. USA.

[19] Machado FB (2020). Systematic analysis of 1298 RNA-Seq samples and construction of a comprehensive soybean (Glycine max) expression atlas. Plant J..

[20] Gonzalez E, Solano R, Rubio V, Leyva A, Paz-Ares J (2005). PHOSPHATE TRANSPORTER TRAFFIC FACILITATOR1 is a plant-specific SEC12-related protein that enables the endoplasmic reticulum exit of a high-affinity phosphate transporter in Arabidopsis. Plant Cell.

[21] Chen JY (2011). OsPHF1 regulates the plasma membrane localization of low- and high-affinity inorganic phosphate transporters and determines inorganic phosphate uptake and translocation in rice. Plant Physiol..

[22] Chen JY (2015). The rice CK2 kinase regulates trafficking of phosphate transporters in response to phosphate levels. Plant Cell.

[23] Nelson BK, Cai X, Nebenfuhr A (2007). A multicolored set of in vivo organelle markers for co-localization studies in Arabidopsis and other plants. Plant J..

[24] Qin, L. et al. Functional characterization of 14 Pht1 family genes in yeast and their expressions in response to nutrient starvation in soybean. *PLoS ONE* **7**, e47726 (2012). 10.1371/journal.pone.0047726 PMC3485015 23133521

[25] Niu, R. et al. uORFlight: a vehicle toward uORF-mediated translational regulation mechanisms in eukaryotes. *Database* **2020**, 1–10 (2020). 10.1093/database/baaa007 PMC7068905 32168374

[26] Nyiko T, Sonkoly B, Merai Z, Benkovics AH, Silhavy D (2009). Plant upstream ORFs can trigger nonsense-mediated mRNA decay in a size-dependent manner. Plant Mol. Biol..

[27] Bai J (2020). A protein-independent fluorescent RNA aptamer reporter system for plant genetic engineering. Nat. Commun..

[28] Li YH (2020). Identification of loci controlling adaptation in Chinese soya bean landraces via a combination of conventional and bioclimatic GWAS. Plant Biotechnol. J..

[29] Guo Z (2021). Genetic analyses of lodging resistance and yield provide insights into post-Green-Revolution breeding in rice. Plant Biotechnol. J..

[30] Muchhal US, Pardo JM, Raghothama KG (1996). Phosphate transporters from the higher plant Arabidopsis thaliana. Proc. Natl Acad. Sci. USA.

[31] Nagy R (2006). Differential regulation of five Pht1 phosphate transporters from maize (Zea mays L.). Plant Biol..

[32] Schunmann PH, Richardson AE, Smith FW, Delhaize E (2004). Characterization of promoter expression patterns derived from the Pht1 phosphate transporter genes of barley (Hordeum vulgare L.). J. Exp. Bot..

[33] Ai P (2009). Two rice phosphate transporters, OsPht1;2 and OsPht1;6, have different functions and kinetic properties in uptake and translocation. Plant J..

[34] Mudge SR, Rae AL, Diatloff E, Smith FW (2002). Expression analysis suggests novel roles for members of the Pht1 family of phosphate transporters in Arabidopsis. Plant J..

[35] Wu P, Shou H, Xu G, Lian X (2013). Improvement of phosphorus efficiency in rice on the basis of understanding phosphate signaling and homeostasis. Curr. Opin. Plant Biol..

[36] Mori A (2016). The role of root size versus root efficiency in phosphorus acquisition in rice. J. Exp. Bot..

[37] Wang X (2016). Genetic variation in ZmVPP1 contributes to drought tolerance in maize seedlings. Nat. Genet..

[38] Li N (2019). Natural variation in ZmFBL41 confers banded leaf and sheath blight resistance in maize. Nat. Genet..

[39] Merchante C, Stepanova AN, Alonso JM (2017). Translation regulation in plants: an interesting past, an exciting present and a promising future. Plant J..

[40] Lin YZ (2019). Impacts of uORF codon identity and position on translation regulation. Nucleic Acids Res..

[41] Sega P, Kruszka K, Szewc L, Szweykowska-Kulinska Z, Pacak A (2020). Identification of transcription factors that bind to the 5’-UTR of the barley PHO2 gene. Plant Mol. Biol..

[42] Zhang H, Wang Y, Lu J (2019). Function and evolution of upstream ORFs in eukaryotes. Trends Biochem. Sci..

[43] Wu HW (2022). Noise reduction by upstream open reading frames. Nat. Plants.

[44] Zhang, H. et al. Genome-wide maps of ribosomal occupancy provide insights into adaptive evolution and regulatory roles of uORFs during Drosophila development. *PLoS Biol*. **16**, e2003903 (2018). 10.1371/journal.pbio.2003903 PMC6070289 30028832

[45] Ribone PA, Capella M, Arce AL, Chan RL (2017). A uORF represses the transcription factor AtHB1 in aerial tissues to avoid a deleterious phenotype. Plant Physiol..

[46] Murphy J, Riley JP (1962). A modified single solution method for the determination of phosphate in natural waters. Anal. Chim. Acta.

[47] Chen S, Zhou Y, Chen Y, Gu J (2018). fastp: an ultra-fast all-in-one FASTQ preprocessor. Bioinformatics.

[48] Li H, Durbin R (2009). Fast and accurate short read alignment with Burrows-Wheeler transform. Bioinformatics.

[49] Li H (2009). The sequence Alignment/Map format and SAMtools. Bioinformatics.

[50] McKenna A (2010). The Genome Analysis Toolkit: a MapReduce framework for analyzing next-generation DNA sequencing data. Genome Res..

[51] Okonechnikov K, Conesa A, Garcia-Alcalde F (2016). Qualimap 2: advanced multi-sample quality control for high-throughput sequencing data. Bioinformatics.

[52] Browning BL, Browning SR (2016). Genotype imputation with millions of reference samples. Am. J. Hum. Genet..

[53] Browning SR, Browning BL (2007). Rapid and accurate haplotype phasing and missing-data inference for whole-genome association studies by use of localized haplotype clustering. Am. J. Hum. Genet..

[54] Cingolani P (2012). A program for annotating and predicting the effects of single nucleotide polymorphisms, SnpEff: SNPs in the genome of *Drosophila melanogaster* strain w1118; iso-2; iso-3. Fly.

[55] Yang J, Lee SH, Goddard ME, Visscher PM (2013). Genome-wide complex trait analysis (GCTA): methods, data analyses, and interpretations. Methods Mol. Biol..

[56] Kumar S, Stecher G, Tamura K (2016). MEGA7: molecular evolutionary genetics analysis version 7.0 for bigger datasets. Mol. Biol. Evol..

[57] Yu GC, Smith DK, Zhu HC, Guan Y, Lam TTY (2017). GGTREE: an R package for visualization and annotation of phylogenetic trees with their covariates and other associated data. Methods Ecol. Evol..

[58] Alexander DH, Novembre J, Lange K (2009). Fast model-based estimation of ancestry in unrelated individuals. Genome Res..

[59] Xie W (2015). Breeding signatures of rice improvement revealed by a genomic variation map from a large germplasm collection. Proc. Natl Acad. Sci. USA.

[60] Danecek P (2011). The variant call format and VCFtools. Bioinformatics.

[61] Zhang C, Dong SS, Xu JY, He WM, Yang TL (2019). PopLDdecay: a fast and effective tool for linkage disequilibrium decay analysis based on variant call format files. Bioinformatics.

[62] Yu YM, Ouyang YD, Yao W (2018). shinyCircos: an R/Shiny application for interactive creation of Circos plot. Bioinformatics.

[63] Lippert C (2011). FaST linear mixed models for genome-wide association studies. Nat. Methods.

[64] Purcell S (2007). PLINK: a tool set for whole-genome association and population-based linkage analyses. Am. J. Hum. Genet..

[65] Li MX, Yeung JM, Cherny SS, Sham PC (2012). Evaluating the effective numbers of independent tests and significant p-value thresholds in commercial genotyping arrays and public imputation reference datasets. Hum. Genet..

[66] Peng WT (2020). Magnesium supports nitrogen uptake through regulating NRT2.1/2.2 in soybean. Plant Soil.

[67] Wang XR (2009). Overexpressing AtPAP15 enhances phosphorus efficiency in soybean. Plant Physiol..

[68] Liu S (2020). A VIT-like transporter facilitates iron transport into nodule symbiosomes for nitrogen fixation in soybean. N. Phytol..

[69] Bai M (2020). Generation of a multiplex mutagenesis population via pooled CRISPR-Cas9 in soya bean. Plant Biotechnol. J..

[70] Chen L (2019). A nodule-localized phosphate transporter GmPT7 plays an important role in enhancing symbiotic N2 fixation and yield in soybean. N. Phytol..

[71] Bun-Ya M, Nishimura M, Harashima S, Oshima Y (1991). The PHO84 gene of *Saccharomyces cerevisiae* encodes an inorganic phosphate transporter. Mol. Cell Biol..

[72] Qin L (2012). The high-affinity phosphate transporter GmPT5 regulates phosphate transport to nodules and nodulation in soybean. Plant Physiol..

