# Supplementary material for: A natural uORF variant confers phosphorus acquisition diversity in soybean
Source: Nat Commun. 2022 Jul 1;13:3796. doi: 10.1038/s41467-022-31555-2 (PMC9249851; doi:10.1038/s41467-022-31555-2)
Supplement: Supplementary file 1 — Supplementary Information [file 41467_2022_31555_MOESM1_ESM.pdf]

A natural uORF variant confers phosphorus acquisition diversity in soybean

Guo *et al*.

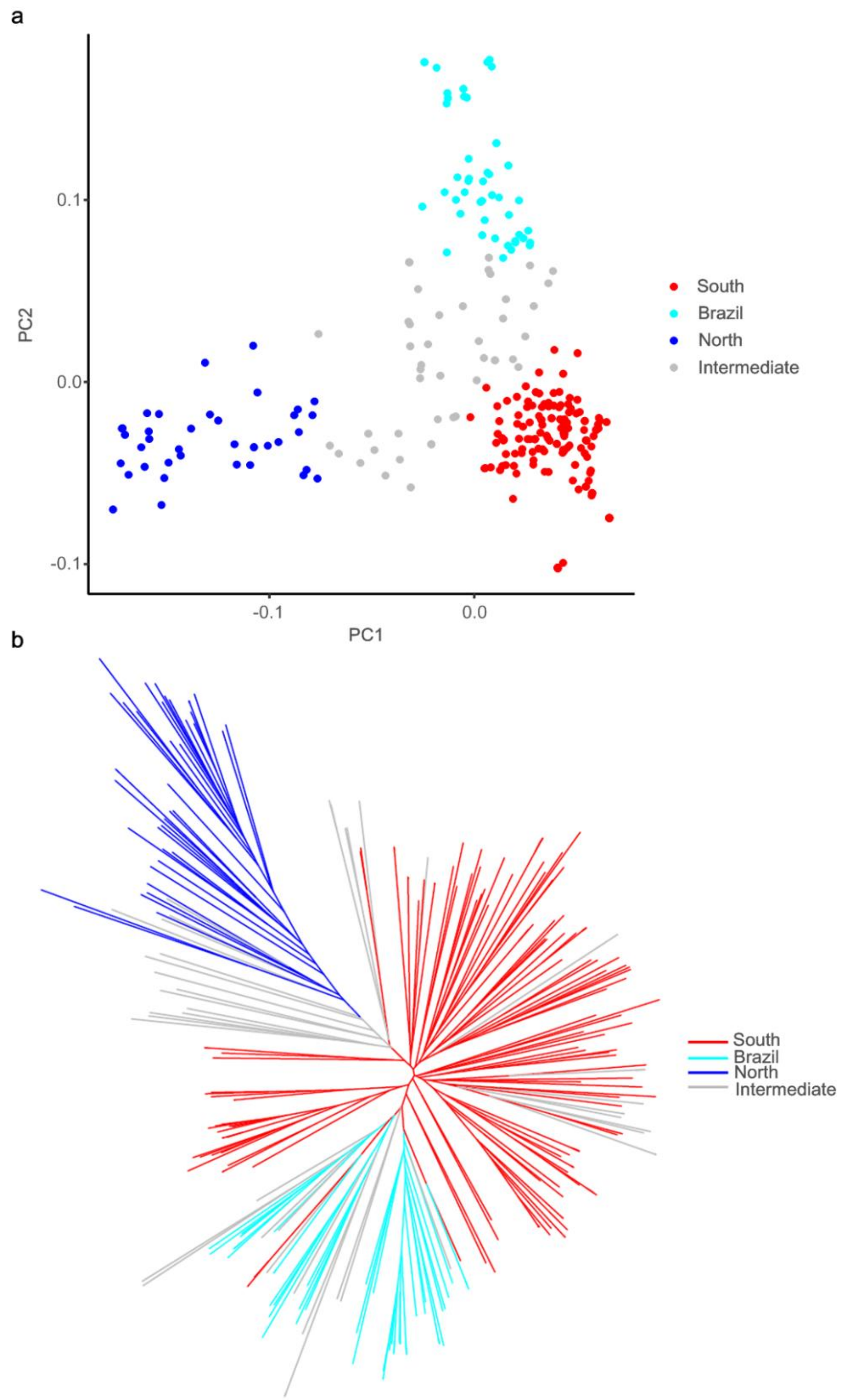

Supplementary Fig. 1. Genetic structure of 274 soybean accessions in the study.

(a) PCA plot of 274 accessions based on genotypes of genome-wide SNPs. PC1 and PC2 represent values of principal component 1 and 2, respectively. Red, cyan, blue, and grey dots indicate accessions from South, Brazil, North subpopulations, and intermediate accessions, respectively.

(b) Neighbor-joining tree of 274 accessions based on genotypes of genome-wide SNPs. Accessions from South, Brazil, North subpopulations, and intermediate accessions are colored red, cyan, blue, and grey, respectively.

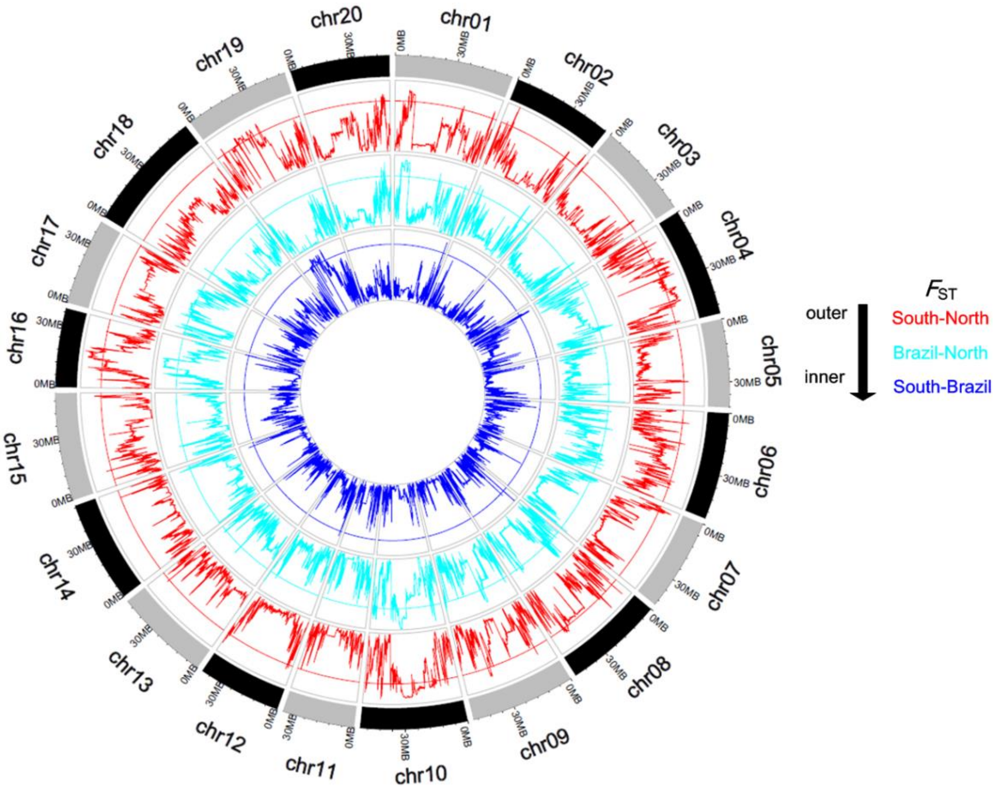

Supplementary Fig. 2. Genome-wide population differentiation statistics (F_{ST}).

F_{ST} levels between South and North subpopulations (red line in outer track), between Brazil and North subpopulations (cyan line in middle track), between South and Brazil subpopulations (blue line in inner track) are shown. Circle in each track indicates the F_{ST} thresholds (5% top F_{ST}).

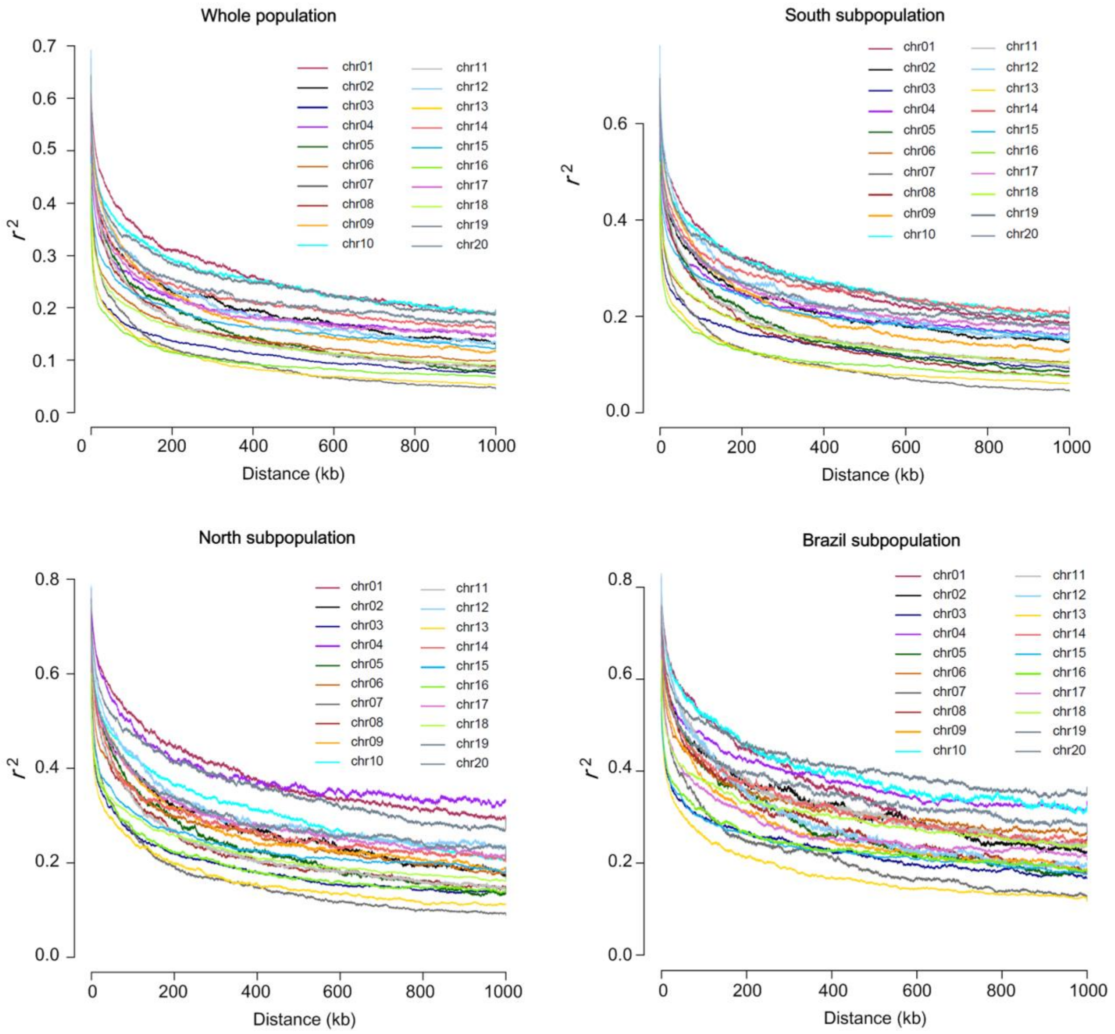

Supplementary Fig. 3. Chromosome-wide LD decay rates.

LD decay rates of different chromosomes are shown in the whole population, South subpopulation, North subpopulation, and Brazil subpopulation, respectively.

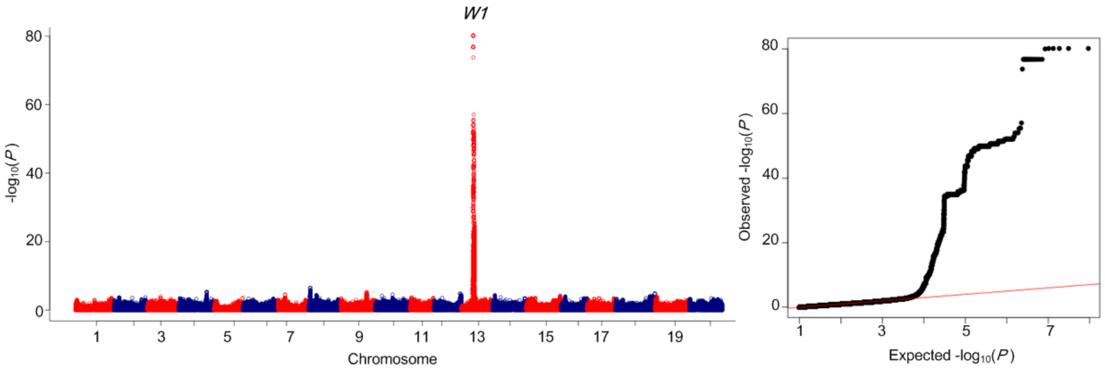

Supplementary Fig. 4. GWAS plots of flower color in the whole population using Linear Mixed Model (LMM). For Manhattan plot (left panel), negative $\log_{10} P$ values are plotted against the position of SNPs on each of 20 chromosomes; for quantile-quantile plot (right panel), the horizontal and vertical axes are the expected negative $\log_{10} P$ values and the observed negative $\log_{10} P$ values, respectively. P values were calculated based on linear mixed model in GWAS and the genome-wide significance was determined with adjustment for multiple tests by the Bonferroni correction.

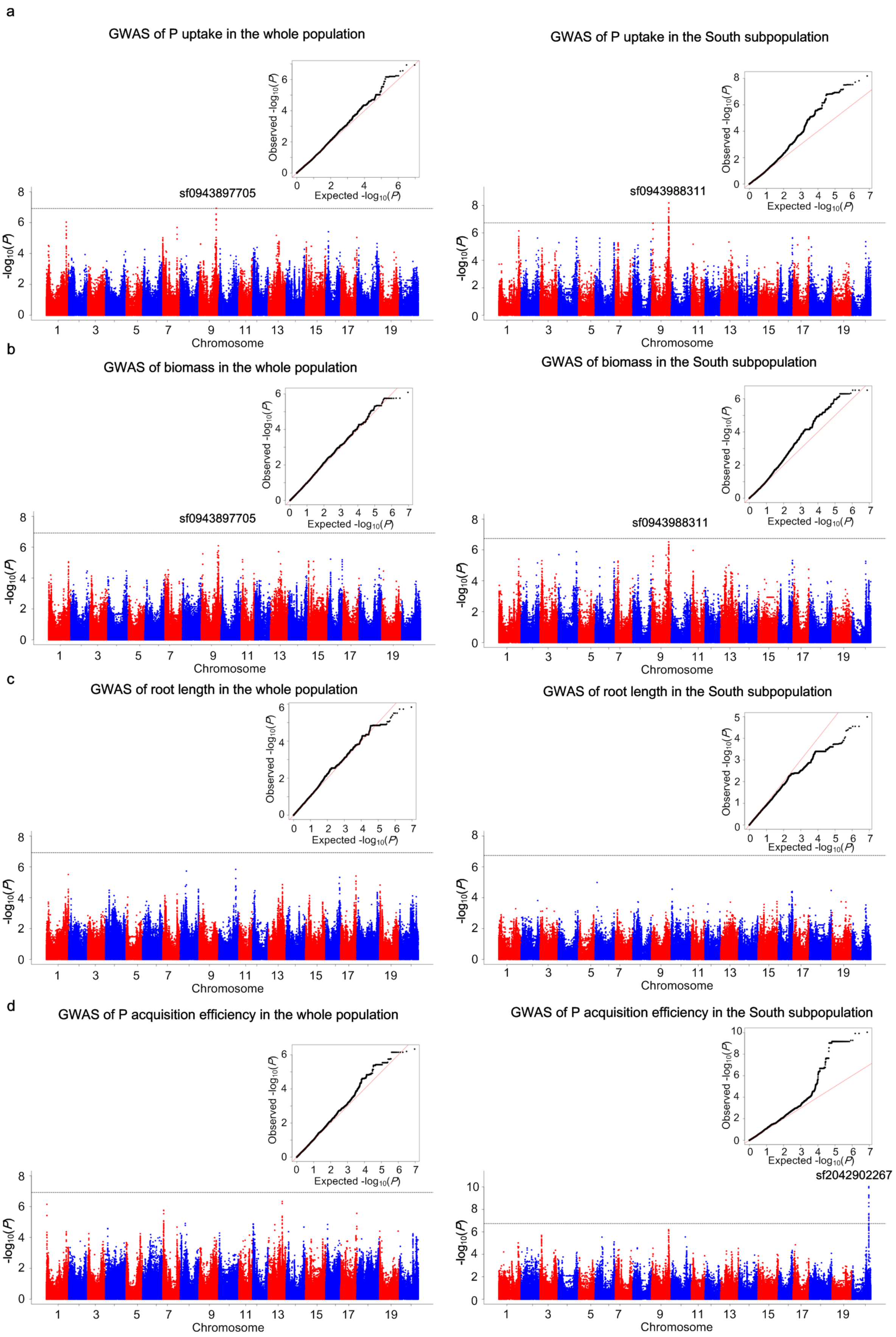
a
GWAS of P uptake in the whole population
GWAS of P uptake in the South subpopulation
sf0943897705
sf0943988311
b
GWAS of biomass in the whole population
GWAS of biomass in the South subpopulation
sf0943897705
sf0943988311
c
GWAS of root length in the whole population
GWAS of root length in the South subpopulation
d
GWAS of P acquisition efficiency in the whole population
GWAS of P acquisition efficiency in the South subpopulation
sf2042902267
Observed -log10(P)
Expected -log10(P)
-log10(P)
Chromosome

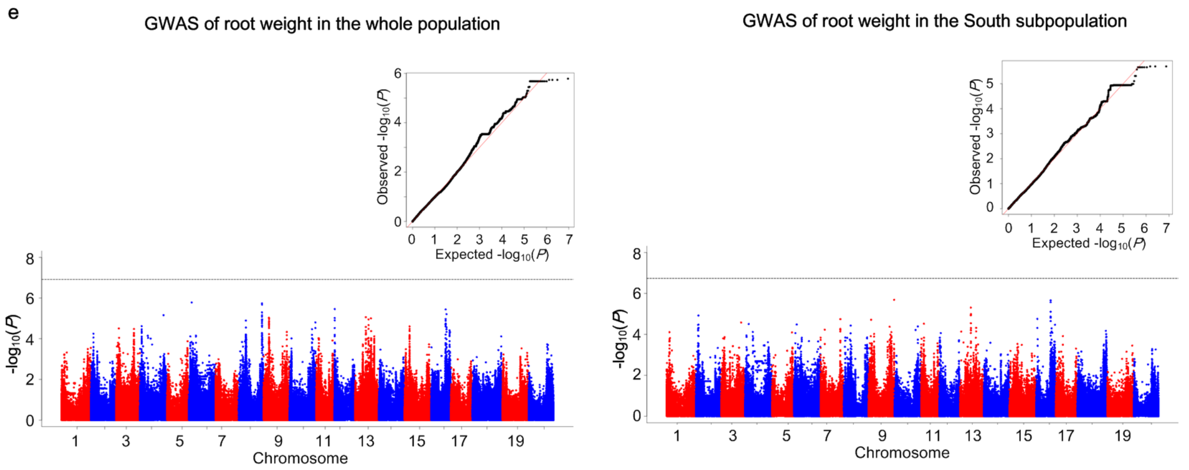

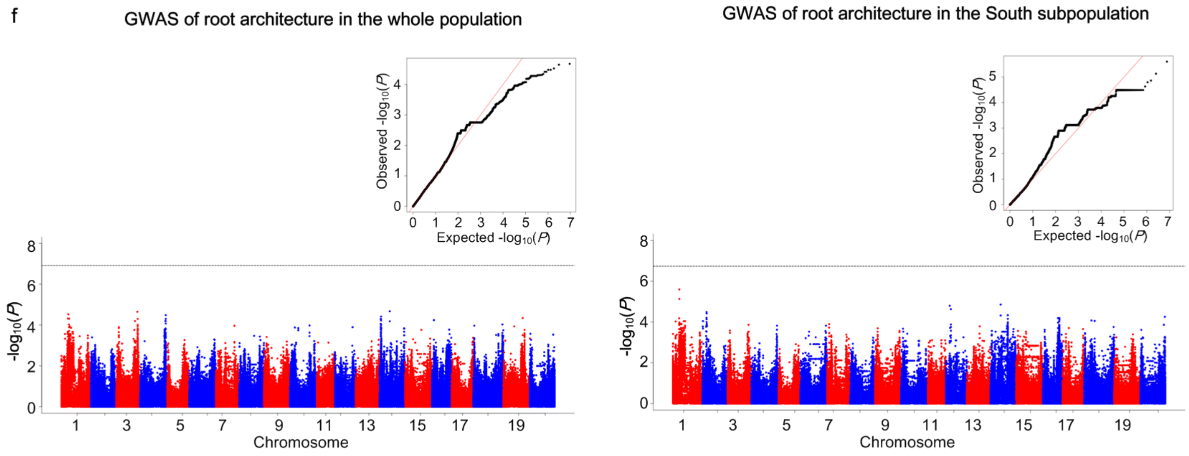

Supplementary Fig. 5. GWAS plots of P-efficiency related traits.

Manhattan plots and quantile-quantile plots of P uptake (**a**), biomass (plant dry weight) (**b**), total root length (**c**), P acquisition efficiency (P uptake per unit root length) (**d**), root weight (**e**), root architecture (**f**) in the whole population and the South subpopulation.

For Manhattan plot, $-\log_{10} P$ values are plotted against the position of SNPs on 20 chromosomes. Grey horizontal dashed line indicates genome-wide *P* value threshold. The lead SNPs are shown above the corresponding association signals. For quantile-quantile plot, $-\log_{10}$-transformed observed *P* values are plotted against $-\log_{10}$-transformed expected *P* values.

In (**a**), SNP sf0943897705 and sf0943988311 were in high LD with each other (LD statistic $r^2 = 0.85$).

In (**f**), the shallow, intermediate, and deep root architecture was scored as 1, 2, 3, respectively. *P* values were calculated based on linear mixed model in GWAS and the genome-wide significance was determined with adjustment for multiple tests by the Bonferroni correction.

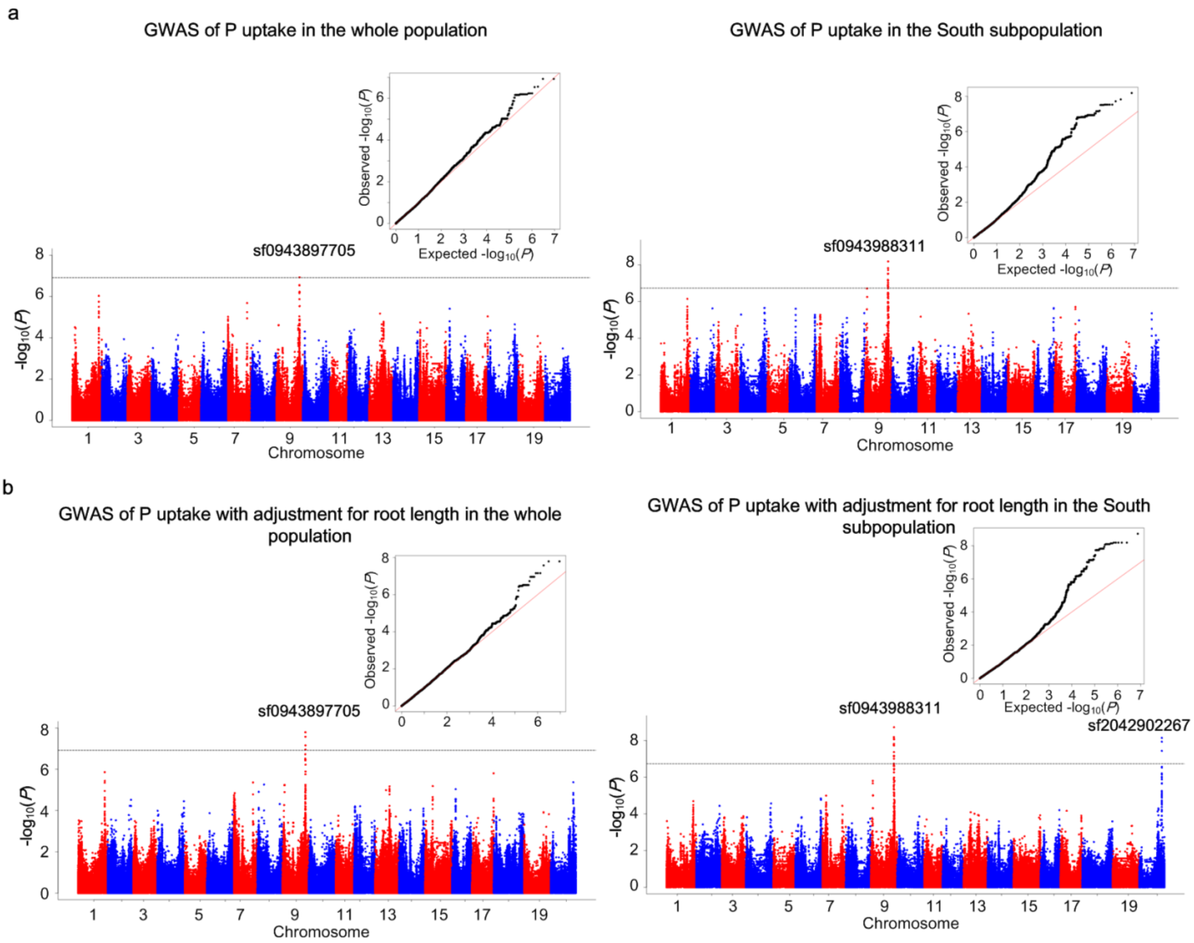

Supplementary Fig. 6. GWAS plots of P uptake with and without adjustment for total root length.

Manhattan plot and quantile-quantile plot of P uptake (**a**) and of P uptake with adjustment for total root length (**b**) in the whole population and the South subpopulation.

For Manhattan plot, $-\log_{10}P$ values are plotted against the position of SNPs on 20 chromosomes. Grey horizontal dashed line indicates genome-wide significance threshold. The lead SNPs are shown above the corresponding association signals. For quantile-quantile plot, $-\log_{10}$-transformed observed P values are plotted against $-\log_{10}$-transformed expected P values. P values were calculated using linear mixed model in GWAS and the genome-wide significance was determined with adjustment for multiple tests by the Bonferroni correction.

SNP sf0943897705 and sf0943988311 were in high LD with each other (LD statistic $r^2 = 0.85$).

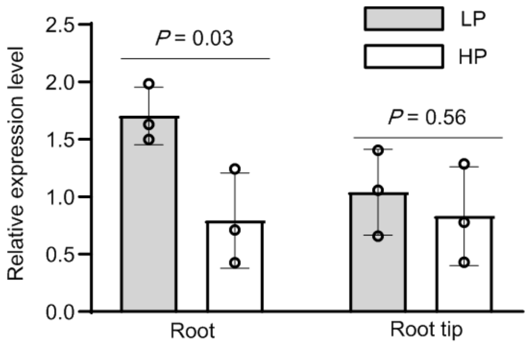

Supplementary Fig. 7. Expression levels of *GmPHF1* in roots in response to P starvation. Soybean plants were grown in hydroponics under low P (LP, 5 μM KH_2PO_4) or normal P conditions (HP, 250 μM KH_2PO_4). Relative expression level quantified by real-time RT-PCR was calculated as the ratio of the expression value of *GmPHF1* to that of *TefS1*. Data represent the mean ± SD from three biological replications. *P* values were calculated based on two-sided *t*-test. Source data underlying Supplementary Fig. 7 are provided as a Source Data file.

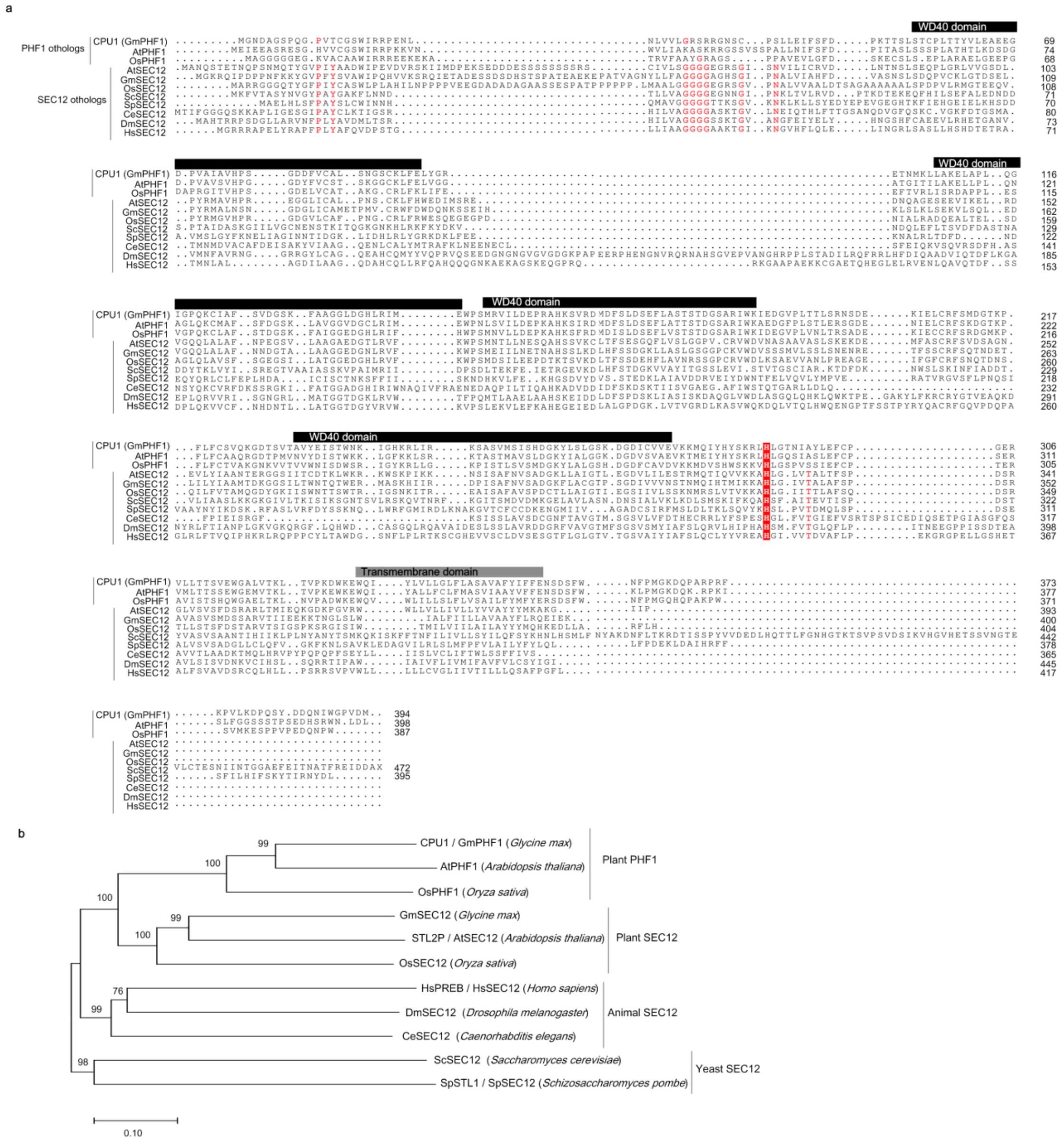

Supplementary Fig. 8. PHF1 orthologs in soybean, *Arabidopsis*, rice, and SEC12 orthologs in plant, animal, yeast.

(a) Peptide sequence alignment for PHF1 and SEC12 orthologs. Red letters indicate the common amino acids among SEC12 orthologs and the letter in the red background indicates the common amino acid among the PHF1 and SEC12 orthologs. The black and grey boxes indicate WD40 domain and transmembrane domain, respectively, which are predicted by SMART (http://smart.embl-heidelberg.de/).

(b) Phylogenetic tree of PHF1 and SEC12 orthologs based on peptide sequences, which was constructed by neighbor-joining tree method of MEGA. The number on the node indicates bootstrap value for 1,000 replications, and the scale at the bottom shows the relationship between branch length and nucleotide substitution rate.

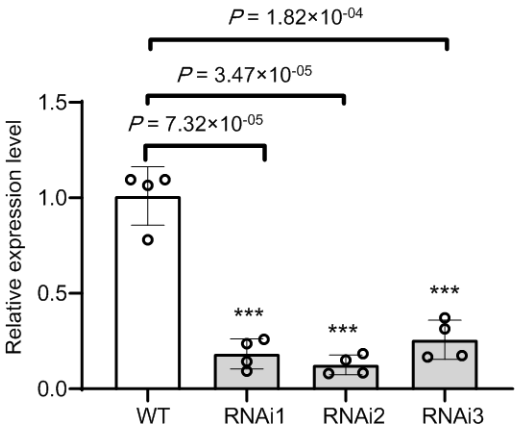

Supplementary Fig. 9. Expression of *GmPHF1* in three independent RNAi lines. The soybean wild type (WT) and three RNAi lines of *GmPHF1* were germinated and grown in vermiculite for 18 days. The true leaves were sampled for RNA isolation and gene expression determination. Data are means ± SD (n = 4 biologically independent samples). *P* values were calculated using two-sided *t*-test. Asterisks indicate significance levels (****P*<0.001). Source data underlying Supplementary Fig. 9 are provided as a Source Data file.

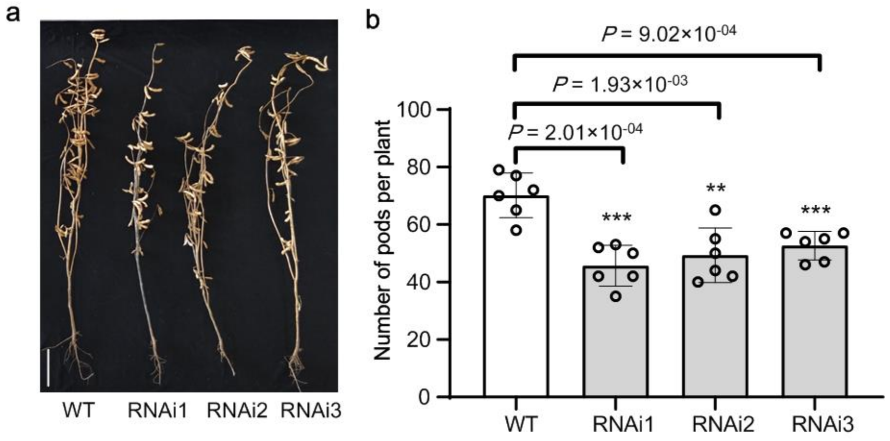

Supplementary Fig. 10. Pod number of WT and three independent RNAi lines of *GmPHF1* grown in the field. (**a**) Performance of WT and three independent RNAi lines grown in the field to the maturity stage (100-d-old plants). Data are presented as mean values ± SD of six biologically independent samples in (**b**). *P* values were calculated using two-sided *t*-test. Asterisks indicate significance levels (***P<0.001, **0.001 ≦ P<0.01). Bar = 10 cm. Source data underlying Supplementary Fig. 10b are provided as a Source Data file.

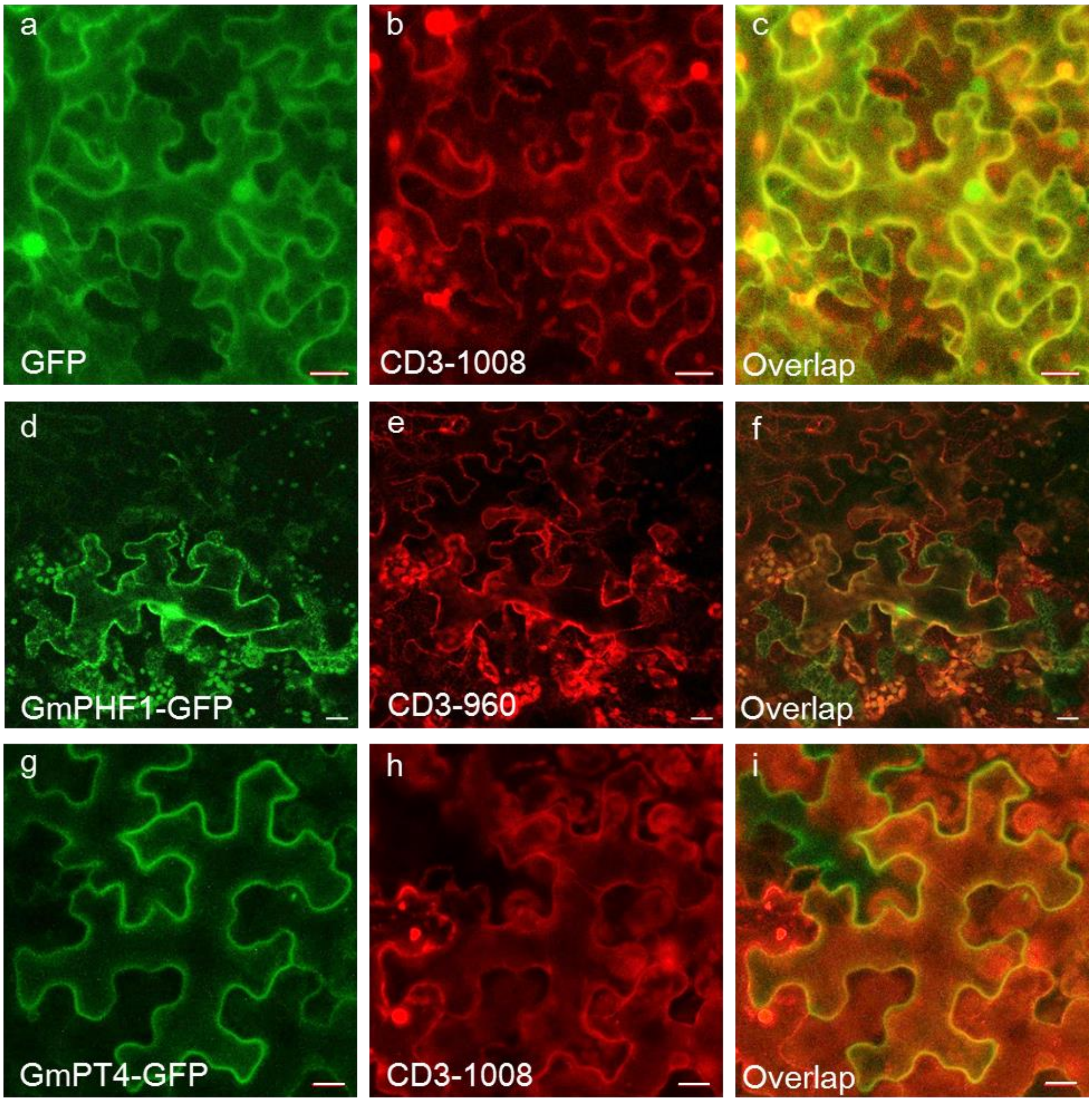

Supplementary Fig. 11. Subcellular localization of GmPHF1 and GmPT4 in tobacco leaves. **a**-**c**, *p35S:GFP*, **d**-**f**, *p35S:GmPHF1-GFP*, **g**-**i**, *p35S:GmPT4-GFP* were co-expressed with PM marker (CD3-1008) or ER marker (CD3-960) fused to mCherry fluorescent protein in tobacco leaves using *Agrobacterium*-mediated transformation. The green fluorescence is the GFP signal (**a**, **d**, **g**), and the red fluorescence is the mCherry signal (**b**, **e**, **h**). Merged images show the combined two channels (**c**, **f**, **i**). Three independent tobacco leaves were investigated and a representative result was shown. bar= 20 μm.

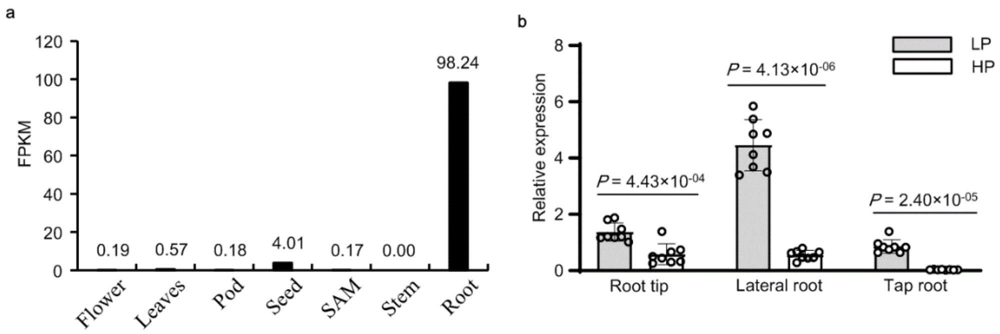

Supplementary Fig. 12. Expression pattern of *GmPT4* in soybean.

(a) FPKM of *GmPT4* in various plant tissues. Data from Phytozome (https://phytozome.jgi.doe.gov). SAM: shoot apical meristem.

(b) Expression pattern of *GmPT4* in response to P deficiency. 4-d-old seedlings were grown in nutrient solution with low P (LP, 5 μM) or high P (HP, 250 μM) for 7 d. Data are presented as means ± SD (n = 8 biologically independent samples). *P* values were calculated using two-sided *t*-test. Source data underlying Supplementary Fig. 12 are provided as a Source Data file.

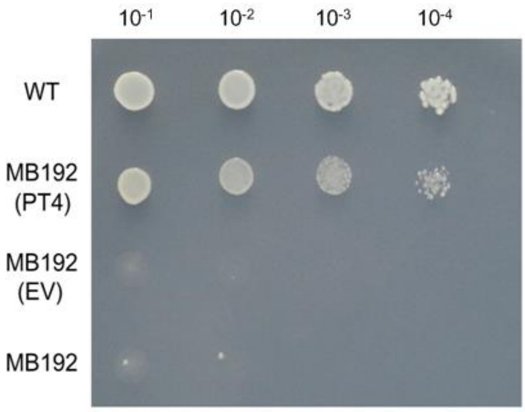

Supplementary Fig. 13. Complementation of Pi transport activity in yeast mutant strain by GmPT4. MB192, a yeast mutant strain defective in high-affinity Pi uptake, was transformed with an empty vector (EV) or a vector expressing GmPT4. WT strain, MB192 and yeast transformants were adjusted to OD600 at 0.1. Ten-fold serial dilutions were spotted onto YNB plates containing 50 μM KH2PO4 at 30°C for 3 d.

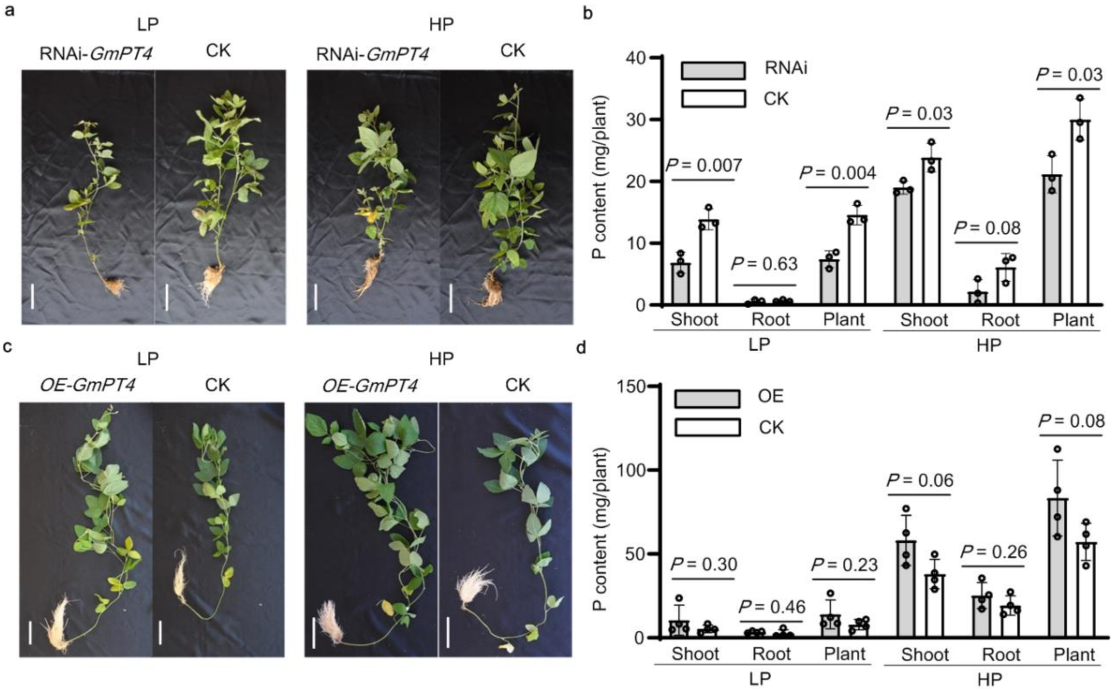

Supplementary Fig. 14. Phenotypic analysis of GmPT4 knockdown and overexpression lines. **a**-**b**, Growth performance (**a**) and P content (**b**) in *GmPT4* knockdown lines. Transgenic (RNAi) and non-transgenic (CK) composite plants were treated with low P (LP, 5 μM) or normal P (HP, 250 μM) for 20 d. Scale bars, 10 cm. Data are presented as means ± SD. n = 3 biologically independent hairy roots.

c-**d**, Growth performance (**c**) and P content (**d**) in *GmPT4* overexpression lines. Transgenic (OE) and non-transgenic (CK) composite plants were treated with low P (LP, 5 μM) or normal P (HP, 250 μM) for 30 d. Scale bars, 10 cm. P concentration was determined by ICP-MS. Data are presented as means ± SD. n = 4 biologically independent hairy roots. *P* values were calculated using two-sided *t*-test. Source data underlying Supplementary Fig. 14b, d are provided as a Source Data file.

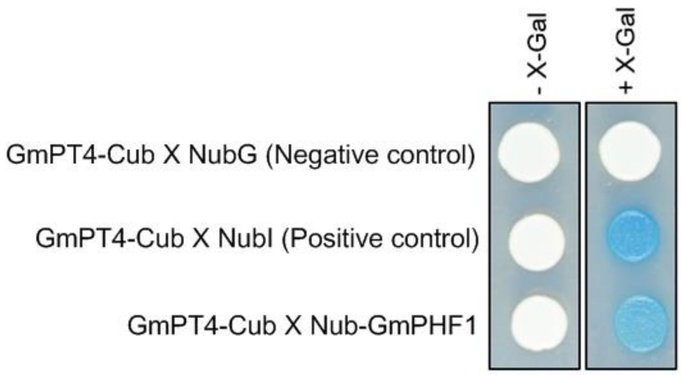

Supplementary Fig. 15. Yeast two-hybrid assay of GmPHF1 and GmPT4. The yeast transformants were spotted onto SD-Leu-Trp medium with or without X-gal for β-galactosidase assay.

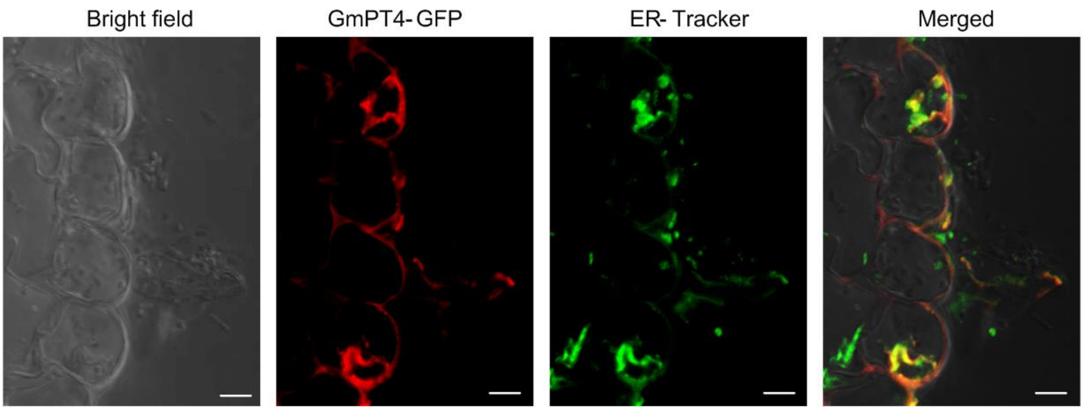

Supplementary Fig. 16. Immunostaining of GmPT4 localization in *GmPHF1*-RNAi line. Immunostaining was performed with *ProPT4: gPT4-GFP* transgenic hairy roots. Red fluorescence is the signal from the GFP antibody and green fluorescence is the signal from the ER marker (ER-Tracker™ Green, Invitrogen). Five independent transgenic lines were investigated and a representative result was shown. Bars = 5 µm.

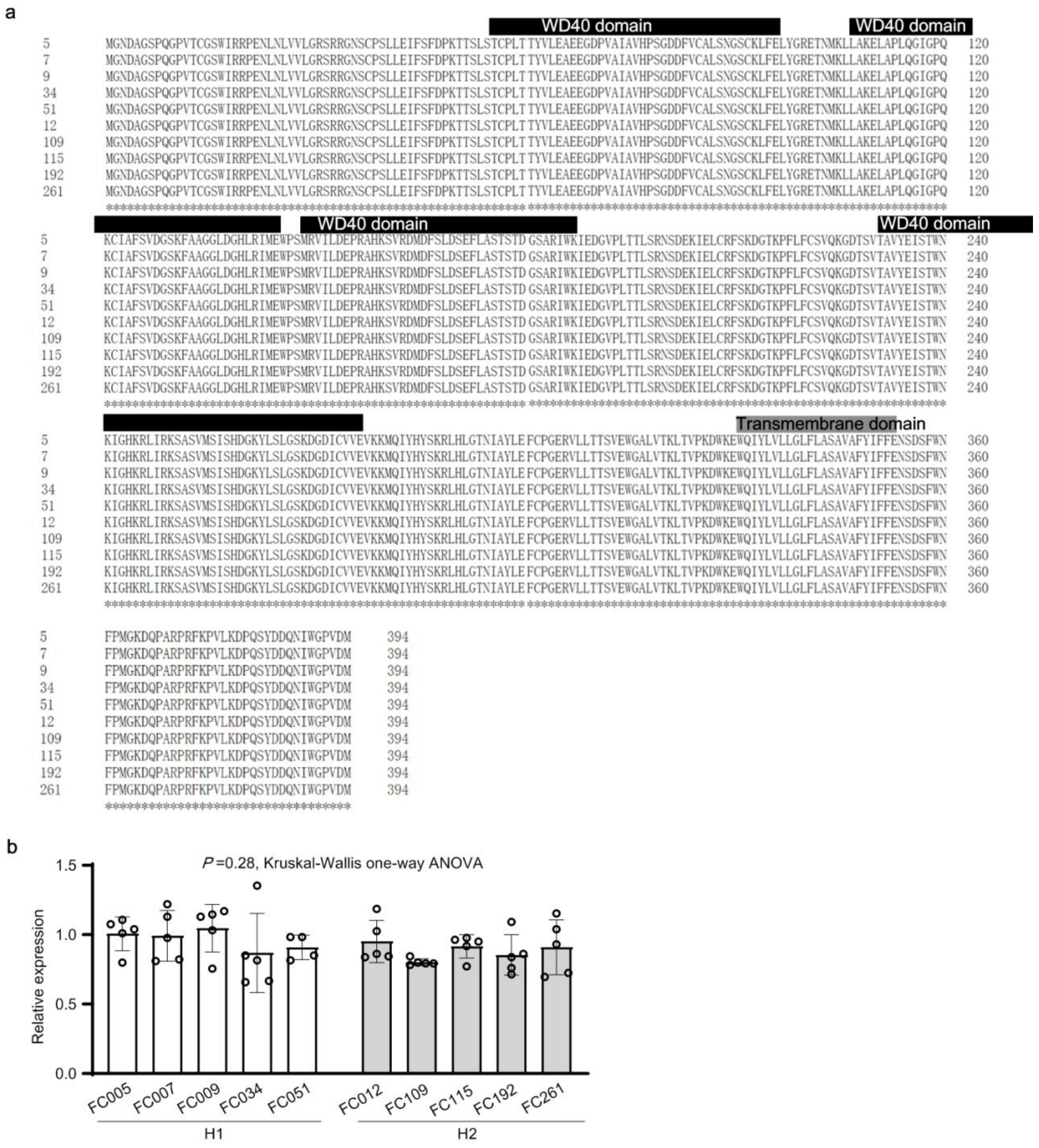

Supplementary Fig. 17. Peptide sequence alignment and expression levels in roots of differnent *GmPHF1*-haplotypes.

(**a**) Peptide sequence alignment of *GmPHF1* for five accessions harboring H1 haplotype and another five accessions harboring H2 haplotype. The black and grey boxes indicate WD40 domain and transmembrane domain, respectively, which are predicted by SMART (http://smart.embl-heidelberg.de/).

(**b**) Expression levels (by real-time RT-PCR) of *GmPHF1* in roots of the ten accessions. The *P* value was calculated using Kruskal-Wallis test. Data are presented as mean values ± SD. n=4 biological independent samples for the soybean accession FC051; n=5 biological independent samples for the other nine accessions. Source data underlying Supplementary Fig. 17b are provided as a Source Data file.

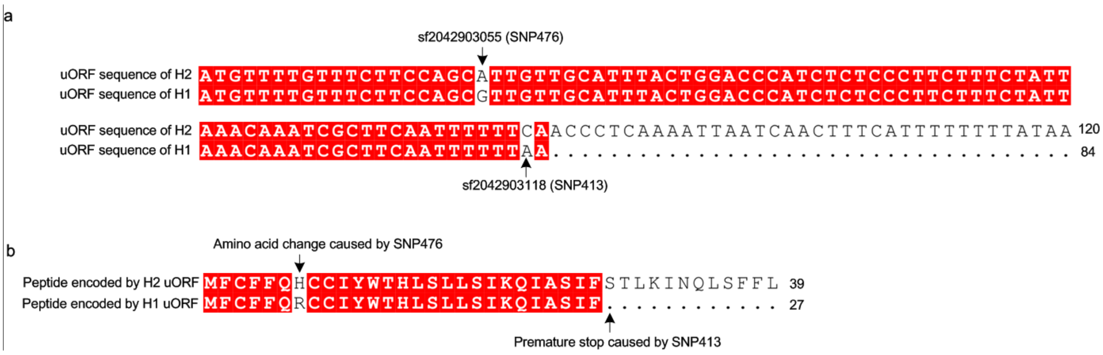

Supplementary Fig. 18. Sequence alignment of uORF of H1 and H2 haplotypes. DNA sequence alignment (**a**) and amino acid sequence alignment (**b**) of uORF of H1 and H2 haplotypes.

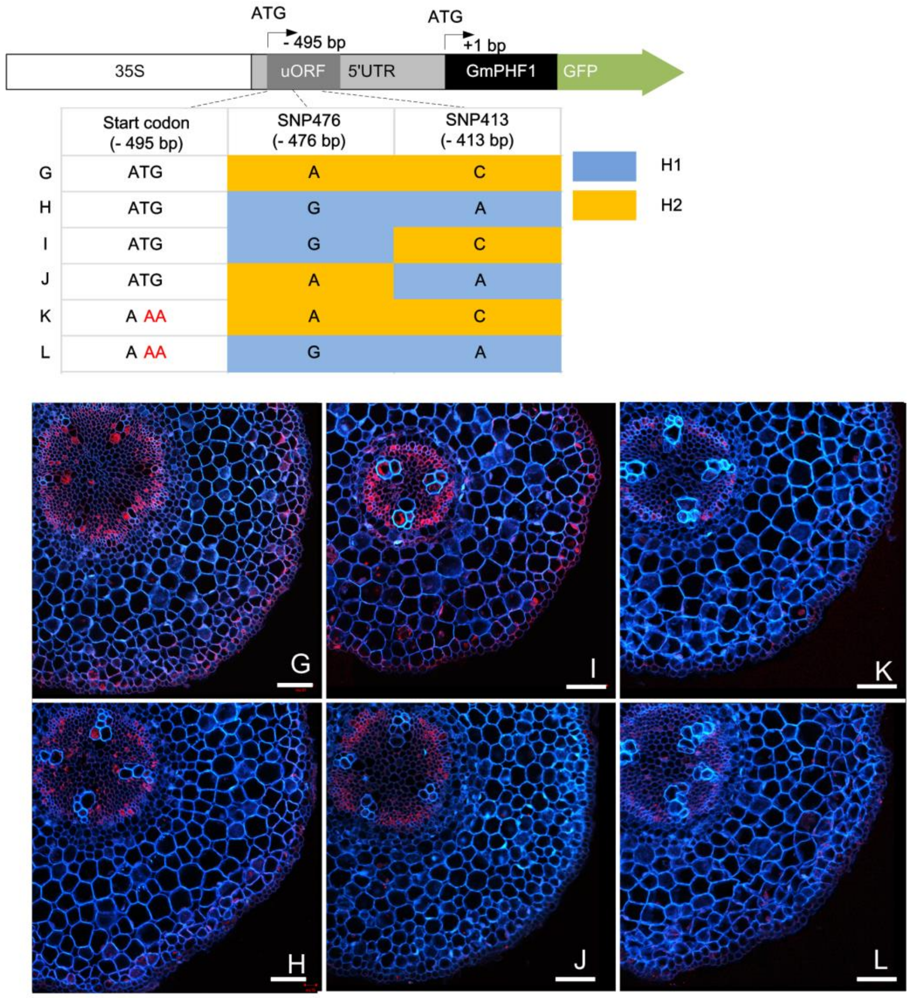

Supplementary Fig. 19. Immunostaining of GmPHF1-GFP in cross sections of transgenic hairy roots carrying the constructs in Fig. 5f. Recombinant constructs (**G-J**) harboring *GmPHF1-GFP* coding region driven by 35S promoter and 5'UTR of different genotypes of naturally occurring SNPs; construct (**K**) and (**L**) harboring H2- and H1-5'UTR with artificial mutation of start codon of uORF (ATG→AAA), respectively. Cyan shows signals from cell walls stained with Calcofluor-White, and red shows the anti-GFP signals. Five independent transgenic lines were investigated and a representative result was shown. Bar= 50 μm.

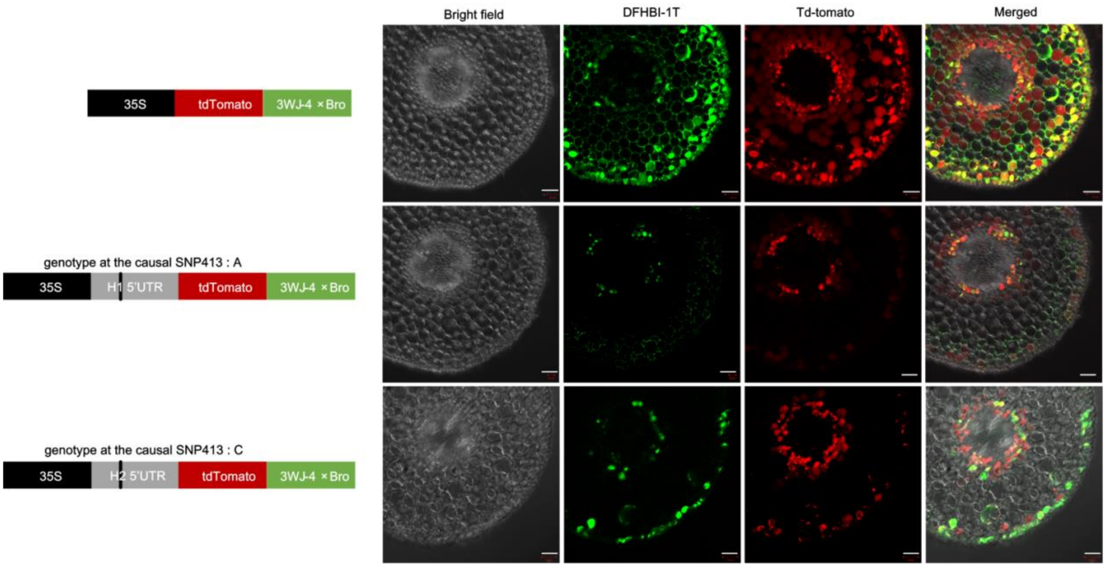

Supplementary Fig. 20. Effect of causal SNP in uORF on mRNA and protein distribution by *in situ* hybridization.

For the constructs, a mRNA reporter 3WJ-4 × Bro is used to visualize tdTomato mRNA distribution (green signals) through DFHBI-1T dye (Lucerna). Red signals indicate the tdTomato protein distribution. Five independent transgenic lines were investigated and a representative result was shown. Bar= 50 μm.

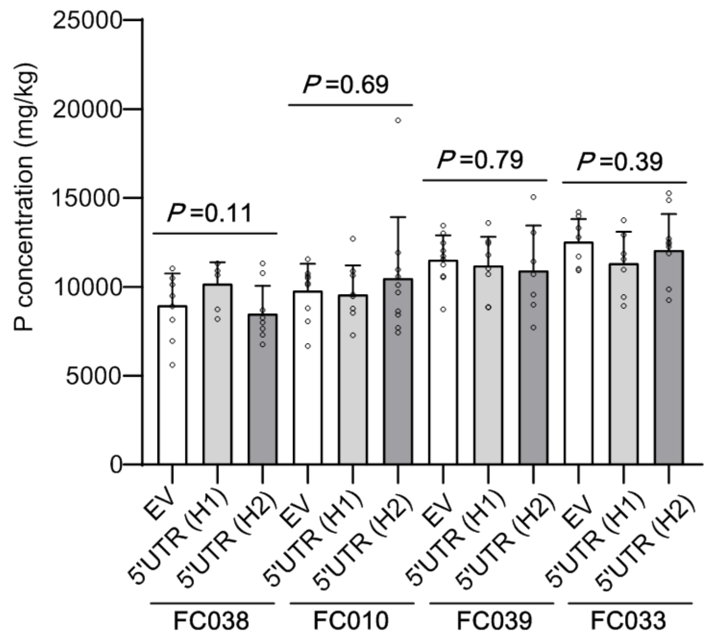

Supplementary Fig. 21. P concentration of transgenic hairy roots of four soybean accessions. promoter (H1)+5'UTR (H1), promoter (H1)+5'UTR (H2) and empty vector were introduced into hairy roots of four soybean accessions (H1 haplotype), respectively. The *P* values were calculated by using one-way ANOVA. Data are presented as mean values±SD. n=9, 7, 9, 10, 9, 10, 10, 9, 7, 9, 7, 10 biological independent samples, respectively. Source data underlying Supplementary Fig. 21 are provided as a Source Data file.

Supplementary Table 1. Summary of SNPs identified in the study.

Chromosome	Length	Number of SNPs	SNP rate	5'UTR	Exon	Intron	Splice site region	3'UTR
chr01	56,831,395	320,147	1 SNP /178 bp	5,732	12,036	35,339	777	5,876
chr02	48,577,505	274,046	1 SNP /177 bp	8,579	15,106	50,305	1,015	7,603
chr03	45,779,434	389,881	1 SNP /117 bp	9,095	17,161	52,241	1,134	8,492
chr04	52,388,848	363,973	1 SNP /144 bp	8,827	15,345	55,710	1,165	8,527
chr05	42,234,498	196,486	1 SNP /215 bp	7,891	12,256	39,645	865	6,586
chr06	51,416,163	376,141	1 SNP /137 bp	12,106	21,315	63,761	1,262	9,187
chr07	44,630,203	252,671	1 SNP /177 bp	11,132	17,857	49,977	1,199	7,898
chr08	47,837,022	273,233	1 SNP /175 bp	12,723	20,243	62,421	1,354	9,516
chr09	50,189,283	289,644	1 SNP /173 bp	9,458	16,056	49,706	1,016	6,894
chr10	51,566,898	282,930	1 SNP /182 bp	7,822	14,583	48,296	1,146	7,001
chr11	34,766,867	185,990	1 SNP /187 bp	6,885	11,596	36,804	909	5,910
chr12	40,091,314	212,050	1 SNP /189 bp	7,286	12,344	36,236	815	6,150
chr13	45,874,162	324,250	1 SNP /141 bp	12,904	22,081	68,375	1,512	11,577
chr14	49,042,192	338,456	1 SNP /145 bp	9,434	15,101	47,146	953	7,937
chr15	51,756,343	447,038	1 SNP /116 bp	10,944	17,658	57,316	1,096	8,666
chr16	37,887,014	358,928	1 SNP /106 bp	13,196	25,270	68,838	1,440	10,005
chr17	41,641,078	258,035	1 SNP /161 bp	7,064	12,678	39,288	1,040	5,998
chr18	58,018,742	565,792	1 SNP /103 bp	14,219	27,738	76,161	1,636	12,045
chr19	50,746,504	319,090	1 SNP /159 bp	7,508	13,720	43,056	913	6,662
chr20	47,900,577	271,613	1 SNP /176 bp	8,010	14,463	50,676	961	6,840
Genome	949,176,042	**6,300,394**	**1 SNP /151 bp**	190,815	334,607	1,031,297	22,208	159,370

Supplementary Table 2. Summary of Insertions/Deletions (INDELs) identified in the study.

Chromosome	Length	Number of insertions	Number of deletions	Number of insertions/deletions (INDELs)	INDEL rate	5'UTR	Exon	Intron	Splice site region	3'UTR
chr01	56,831,395	48,309	52,018	100,327	1 INDEL / 566 bp	3,379	1,568	16,080	390	3,128
chr02	48,577,505	47,402	50,375	97,777	1 INDEL / 497 bp	5,071	2,160	23,881	426	3,675
chr03	45,779,434	57,547	61,649	119,196	1 INDEL / 384 bp	4,834	2,237	24,320	473	4,459
chr04	52,388,848	51,752	55,466	107,218	1 INDEL / 489 bp	4,400	1,694	21,751	441	3,934
chr05	42,234,498	36,587	39,252	75,839	1 INDEL / 557 bp	4,159	1,413	17,424	353	3,257
chr06	51,416,163	59,986	65,084	125,070	1 INDEL / 411 bp	6,260	3,156	27,480	561	4,653
chr07	44,630,203	47,598	49,930	97,528	1 INDEL / 458 bp	6,319	2,543	22,538	470	3,806
chr08	47,837,022	54,234	56,242	110,476	1 INDEL / 433 bp	6,851	2,452	30,070	482	5,419
chr09	50,189,283	52,234	55,492	107,726	1 INDEL / 466 bp	5,452	2,214	23,422	380	3,676
chr10	51,566,898	50,365	52,973	103,338	1 INDEL / 499 bp	4,545	1,817	23,943	385	3,449
chr11	34,766,867	34,906	36,999	71,905	1 INDEL / 484 bp	3,790	1,370	15,913	340	2,876
chr12	40,091,314	36,955	38,171	75,126	1 INDEL / 534 bp	3,478	1,661	16,101	392	3,333
chr13	45,874,162	61,615	65,531	127,146	1 INDEL / 361 bp	7,730	2,976	30,766	533	5,861
chr14	49,042,192	52,293	55,423	107,716	1 INDEL / 455 bp	4,638	2,197	20,301	465	3,587
chr15	51,756,343	59,826	63,925	123,751	1 INDEL / 418 bp	5,366	2,195	22,498	401	3,812
chr16	37,887,014	54,859	61,194	116,053	1 INDEL / 326 bp	6,396	4,094	26,158	606	4,568
chr17	41,641,078	45,316	47,784	93,100	1 INDEL / 447 bp	4,252	1,665	18,887	412	3,113
chr18	58,018,742	78,382	85,386	163,768	1 INDEL / 354 bp	7,055	3,416	30,518	517	5,215
chr19	50,746,504	47,655	51,063	98,718	1 INDEL / 514 bp	4,346	1,820	19,602	351	3,249
chr20	47,900,577	46,124	49,667	95,791	1 INDEL / 500 bp	4,472	1,761	21,351	391	3,510
Genome	949,176,042	1,023,945	1,093,624	**2,117,569**	1 INDEL / 448 bp	102,793	44,409	453,004	8,769	78,580

Supplementary Table 3. Number of effective SNPs calculated by using GEC and genome-wide significance threshold.

Chromosome	Whole population				South subpopulation			
	Observed # of SNPs with MAF ≧ 0.05)	Effective number (Ne)	Effective ratio	Genome-wide sig. threshold (0.05/Ne)	Observed number of SNPs with MAF ≧ 0.05)	Effective number (Ne)	Effective ratio	Genome-wide sig. threshold (0.05/Ne)
chr01	248,330	16,020	0.06		209,003	11,395	0.05	
chr02	186,231	15,815	0.08		175,087	11,619	0.07	
chr03	269,684	30,636	0.11		246,484	22,459	0.09	
chr04	256,812	21,050	0.08		193,849	13,369	0.07	
chr05	128,770	10,609	0.08		104,797	6,673	0.06	
chr06	279,134	27,976	0.10		217,556	16,606	0.08	
chr07	184,579	18,995	0.10		159,773	13,768	0.09	
chr08	216,653	16,952	0.08		145,669	9,059	0.06	
chr09	238,930	18,146	0.08		205,232	12,381	0.06	
chr10	231,657	17,120	0.07		133,910	7,940	0.06	
chr11	141,462	11,954	0.08		122,466	8,838	0.07	
chr12	128,323	8,998	0.07		87,730	4,443	0.05	
chr13	229,920	27,542	0.12		190,093	18,286	0.10	
chr14	191,214	16,244	0.08		203,047	13,525	0.07	
chr15	359,179	31,874	0.09		302,283	20,529	0.07	
chr16	248,610	31,040	0.12		207,405	21,925	0.11	
chr17	214,308	16,186	0.08		182,755	10,369	0.06	
chr18	424,108	43,822	0.10		361,846	29,576	0.08	
chr19	240,295	16,053	0.07		173,834	9,808	0.06	
chr20	200,675	17,026	0.08		134,878	9,319	0.07	
**Genome**	4,618,874	414,058	0.09	**1.21E-07**	3,757,697	271,887	0.07	**1.84E-07**

Supplementary Table 4. Soil chemical properties in the experimental field site.

pH	Organic matter (g/kg)	Total N (g/kg)	Total P (g/kg)	Total K (g/kg)	Available N (mg/kg)	Available P (mg/kg)			Available K (mg/kg)
						0-20 cm	20-40 cm	40-60 cm	
5.91	15.73	0.93	0.55	15.32	87.39	23.79	4.95	0.19	91.32
