# Supplementary material for: A natural uORF variant confers phosphorus acquisition diversity in soybean
Source: Nat Commun. 2022 Jul 1;13:3796. doi: 10.1038/s41467-022-31555-2 (PMC9249851; doi:10.1038/s41467-022-31555-2)
Supplement: Supplementary file 2 — Description of Additional Supplementary Files [file 41467_2022_31555_MOESM2_ESM.pdf]

Description of Additional Supplementary Files

File Name: Supplementary Data 1
Description: Detailed information of 274 accessions in an applied core collection of P-efficiency in soybean

File Name: Supplementary Data 2
Description: Divergent regions between subpopulations detected by FST analysis

File Name: Supplementary Data 3
Description: GWAS results of the study

File Name: Supplementary Data 4
Description: Expression profiles of annotated genes of CPU1 locus.

File Name: Supplementary Data 5
Description: Primers used in the study
