# Supplementary figures and images for: A natural uORF variant confers phosphorus acquisition diversity in soybean
Source: Nat Commun. 2022 Jul 1;13:3796. doi: 10.1038/s41467-022-31555-2 (PMC9249851; doi:10.1038/s41467-022-31555-2)

Figure 5d

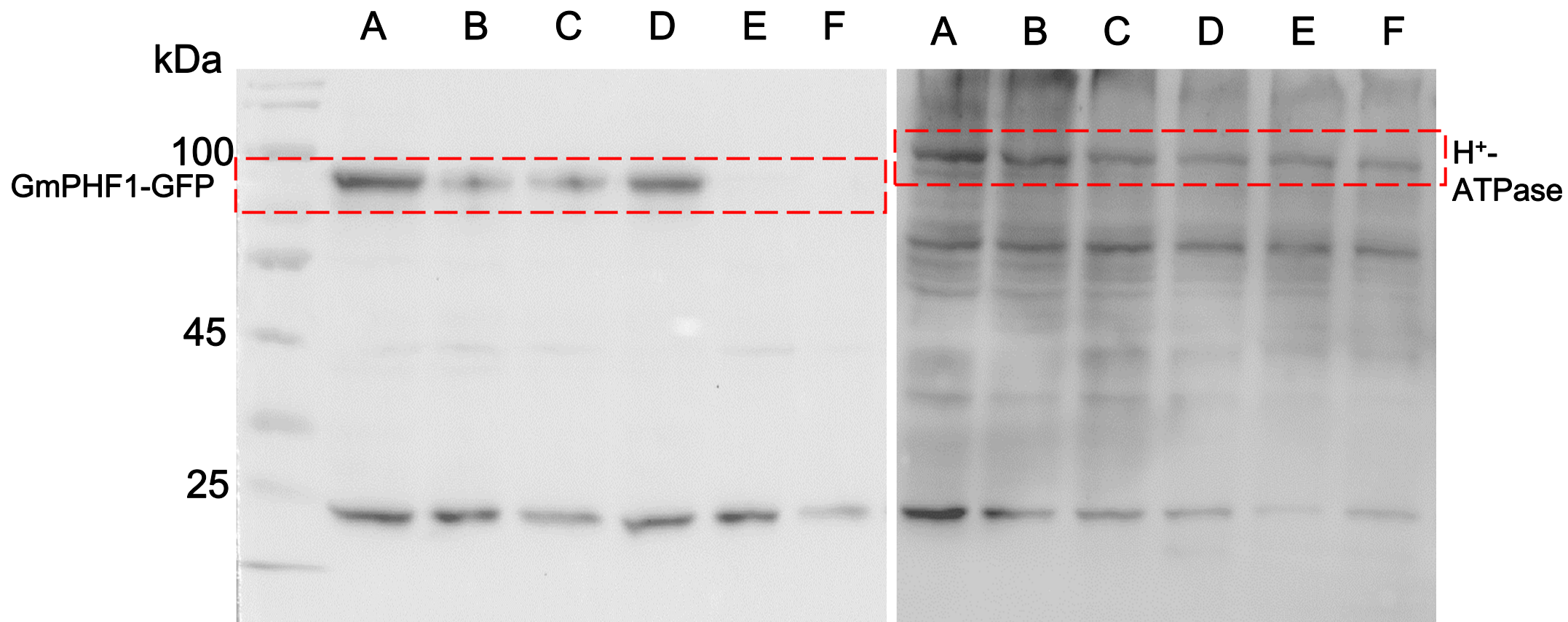

Figure 5g

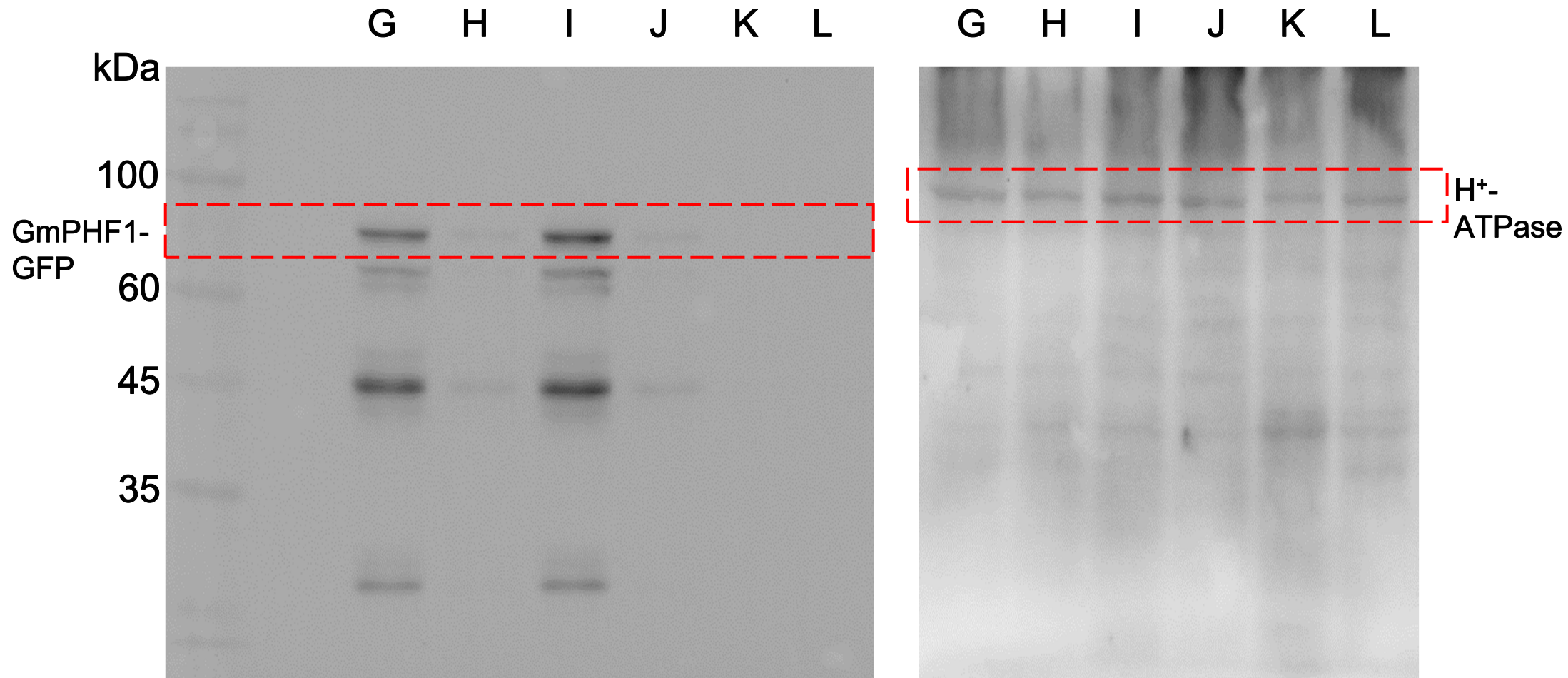

Supplement: Supplementary file 9 — Source Data [file 41467_2022_31555_MOESM9_ESM.zip › Fig. 5d and 5g_Uncropped scans of Western-blots.pdf]
